# Pharmacokinetic-Modifying Linker Design Modulates Performance of Multivalent PSMA/GRPR-Cell-Targeting ^177^Lu Radiopharmaceuticals

**DOI:** 10.3390/cancers18203252

**Published:** 2026-10-09

**Authors:** Claudia G. Chambers, Grace Liles, Anupam Mathur, Tamer M. Sakr, John M. Himmelberg, Fabio Gallazi, Lisa Watkinson, Terry Carmack, Yue Guan, Susan Rottinghaus, Yubin Miao, Christine Lovingier, Thomas Collora, Gracie Miller, Michael R. Lewis, John D. Brockman, Carolyn J. Anderson, Charles J. Smith

**Affiliations:** 1 Department of Chemistry, University of Missouri, Columbia, MO 65211, USA; cgcgh4@umsystem.edu (C.G.C.); gkliles@uab.edu (G.L.); jmhcxf@mail.missouri.edu (J.M.H.); cllx8n@missouri.edu (C.L.); carolyn.j.anderson@missouri.edu (C.J.A.); 2 Research Division, Harry S. Truman Memorial Veterans’ Hospital, Columbia, MO 65201, USA; tamer.sakr@fulbrightmail.org (T.M.S.); watkinsonl@health.missouri.edu (L.W.); carmackte@health.missouri.edu (T.C.); tc8dd@missouri.edu (T.C.IV); gkmfy6@umsystem.edu (G.M.); 3 Molecular Imaging and Theranostics Center, University of Missouri, Columbia, MO 65211, USA; amathur@britatom.gov.in (A.M.); brockmanjd@missouri.edu (J.D.B.); 4 Radioactive Isotopes and Generator Department, Hot Labs Center, Egyptiona Atomic Energy Authority, Cairo 13759, Egypt; 5 Molecular Interactions Core, University of Missouri, Columbia, MO 65211, USA; gallazzif@missouri.edu; 6 Department of Molecular Microbiology and Immunology, Bond Life Sciences Center, School of Medicine, University of Missouri, Columbia, MO 65212, USA; guanyue@missouri.edu; 7 Cell and Immunobiology Core, University of Missouri, Columbia, MO 65211, USA; rottinghauss@health.missouri.edu; 8 Department of Radiology and Stony Brook Cancer Center, Renaissance School of Medicine, Stony Brook, NY 11794, USA; yubin.miao@stonybrookmedicine.edu; 9 Department of Veterinary Medicine and Surgery, College of Veterinary Medicine, University of Missouri, Columbia, MO 65211, USA; lewismic@missouri.edu; 10 University of Missouri Research Reactor, University of Missouri, Columbia, MO 65211, USA; 11 Department of Radiology, School of Medicine, University of Missouri, Columbia, MO 65212, USA; 12 Ellis Fischel Cancer Center, School of Medicine, University of Missouri, Columbia, MO 65212, USA

**Keywords:** prostate cancer, heterobivalent, theranostics, PSMA, GRPR, lutetium-177, DOTA, SPECT/CT, dual-targeting

## Abstract

**Background/Objectives**: A series of PSMA/GRPR-targeting compounds were selected based on modifications to their respective pharmacokinetic-modifying linking groups and the structure of the PSMA-binding moiety, to examine how these alterations impact binding affinity and specificity for preclinical prostate cancer models. **Methods**: The multi-receptor targeting heterodimers PSMA/2PSMA-X-DOTA-Y-BBN (where X is 6-aminohexanoic acid (6AHX) or polyethylene glycol-4 (PEG_4_) and Y is either 8-aminooctanoic acid (8AOC) or carboxy piperidine (CP)) were radiolabeled with the theranostic isotope [^177^Lu]Lu. Each heterodimer construct was evaluated through characterization, stability, whole-cell in vitro studies, biodistribution in tumor-bearing mice, and in vivo small animal SPECT/CT molecular imaging. **Results**: In vitro evaluation of 2PSMA-6AHX-DOTA-8AOC-BBN demonstrated moderate IC_50_ values of 200 ± 20 nM in LNCaP cells and 21 ± 1 nM in PC3 cells. Subsequent biodistribution studies demonstrated selective tumor uptake at 4 h post-intravenous injection with uptake of 9.52 ± 2.59%ID/g in GRPR[+] PC3 tumors and 6.37 ± 0.64%ID/g in PSMA[+] PC3-PIP tumors. At 24 h post-injection, high tumor-to-blood ratios (874.0 ± 276 in PC3 and 344.3 ± 85 in PC3-PIP) and tumor-to-muscle ratios (739.8 ± 277 in PC3 and 403.5 ± 66 in PC3-PIP) were observed, further supporting the theranostic feasibility of the radiolabeled heterodimer. Small animal SPECT/CT imaging confirmed tumor targeting specificity of the heterobivalent peptide. **Conclusions**: Evaluation in PSMA/GRPR-expressing tumor models identified 2PSMA-6AHX-DOTA-8AOC-BBN as a leading candidate, having enhanced tumor uptake and favorable pharmacokinetics in tumor-bearing mice. These investigations advance structure–activity relationship understanding and support heterobivalent radiopharmaceuticals for precision imaging and targeted prostate cancer therapy.

## Simple Summary

Prostate cancer remains a leading cause of cancer-related morbidity, driving the need for improved diagnostic and therapeutic tools for proper intervention in patients. Hybrid or multivalent radiopharmaceuticals, which target single- or multi-targetable receptors simultaneously, offer a promising strategy to enhance tumor specificity and imaging contrast. The primary objectives of this work are to (1) optimize the pharmacokinetic properties of the prostate-specific membrane antigen (PSMA)- and gastrin-releasing peptide receptor (GRPR) heterobivalent scaffold through modifications to pharmacokinetic modifying linking groups to enhance binding efficiency, (2) evaluate the radiochemical stability of DOTA-conjugated agents labeled with [^177^Lu], and (3) examine their tissue uptake and tumor targeting capacity through biodistribution investigations and small animal imaging studies in relevant preclinical animal models.

## 1. Introduction

Prostate cancer remains a leading cause of morbidity and mortality among men worldwide, with disease progression often accompanied by significant molecular heterogeneity. In 2026 alone, it is projected that the United States will have 333,830 new cases of prostate cancer and 36,320 deaths [1].

To combat the global cancer burden, theranostic radiopharmaceuticals have quickly become a cornerstone of modern cancer care in oncology, particularly for neuroendocrine and prostate cancer tumors. For example, market-available radiopharmaceuticals that target prostate cancer biomarkers, such as the prostate-specific membrane antigen (PSMA), have demonstrated substantial clinical impact in recent years. Drugs such as the gallium diagnostic agent [^68^Ga]Ga-PSMA-11 (Locametz^®^) and the targeted radiotherapy [^177^Lu]Lu-PSMA-617 (Pluvicto^®^) [2,3,4,5] target this cell-surface antigen. However, variable PSMA expression across disease stages and between metastatic lesions can limit sensitivity and therapeutic efficacy [6,7,8,9,10,11]. Gastrin-releasing peptide receptor (GRPR) presents as a complementary molecular target that is frequently over-expressed on prostate cancer cells, particularly in earlier-stage and PSMA low-grade tumors [12,13,14,15,16]. Strategies capable of addressing this receptor heterogeneity are therefore of considerable interest in improving both detection and treatment of this disease. Multi receptor-targeting hybrid or heterodimeric radiopharmaceuticals offer distinct advantages over the administration of monovalent agents, such as improved tumor uptake and retention, greater tumor specificity, receptor avidity, and simplified clinical translation [17,18,19,20,21]. By integrating more than one targeting motif within a single molecular framework, heterodimers may improve tumor localization across heterogeneous lesions, enabling the ability to reach a larger cohort of patients.

Dual-targeting PSMA/GRPR- heterodimers represent a promising strategy for improving prostate cancer detection and therapy, by taking advantage of complementary receptor expression profiles that are often detected in a majority of PCa cases [22]. Previous work in our laboratory by Bandari et al. established the design and feasibility of the PSMA/GRPR-targeting heterodimer, PSMA-6AHX-DOTA-8AOC-BBN [23]. This peptide demonstrated favorable in vivo tumor uptake and imaging performance in PSMA[+] and GRPR[+] tumor models [20,23,24].

These findings supported further investigation of the scaffold, particularly with respect to tumor uptake and targeting properties. Building on this work, our group evaluated modifications of the PSMA/GRPR- dual-targeting scaffold to improve biological uptake. Linker design becomes particularly important in more complex constructs such as dual-targeting heterodimeric radiopharmaceuticals, where spatial orientation and flexibility can significantly influence receptor engagement and pharmacokinetics. As such, a rational and comparative assessment of linker flexibility, hydrophobicity, and targeting moiety structure is necessary for optimizing dual-receptor cell-targeting radiopharmaceuticals. Various pharmacokinetic modifying linking groups have been incorporated in the heterodimer constructs to build upon previous work to evaluate their influence on molecular flexibility, pharmacokinetics, and cell-surface receptor engagement. The hydrophobic linkers 6-aminohexanoic acid (6AHX) and 8-aminooctanoic acid (8AOC), originally evaluated in our laboratory, were incorporated into a PSMA-6AHX-DOTA-8AOC-BBN scaffold, where BBN = bombesin(7–14)NH_2_. The linkers moderate rigidity through their linear aliphatic structure, while retaining some flexibility along the carbon chain [23,24,25]. Carboxymethyl piperine (CP), a more rigid heterocyclic linker previously used in the GRPR antagonist DOTA-RM2, has also been evaluated [26,27,28,29]. The hydrophilic spacer polyethylene glycol-4 (PEG_4_) was also evaluated for its ability to improve solubility and reduce nonspecific interaction [30].

**Figure 1:**
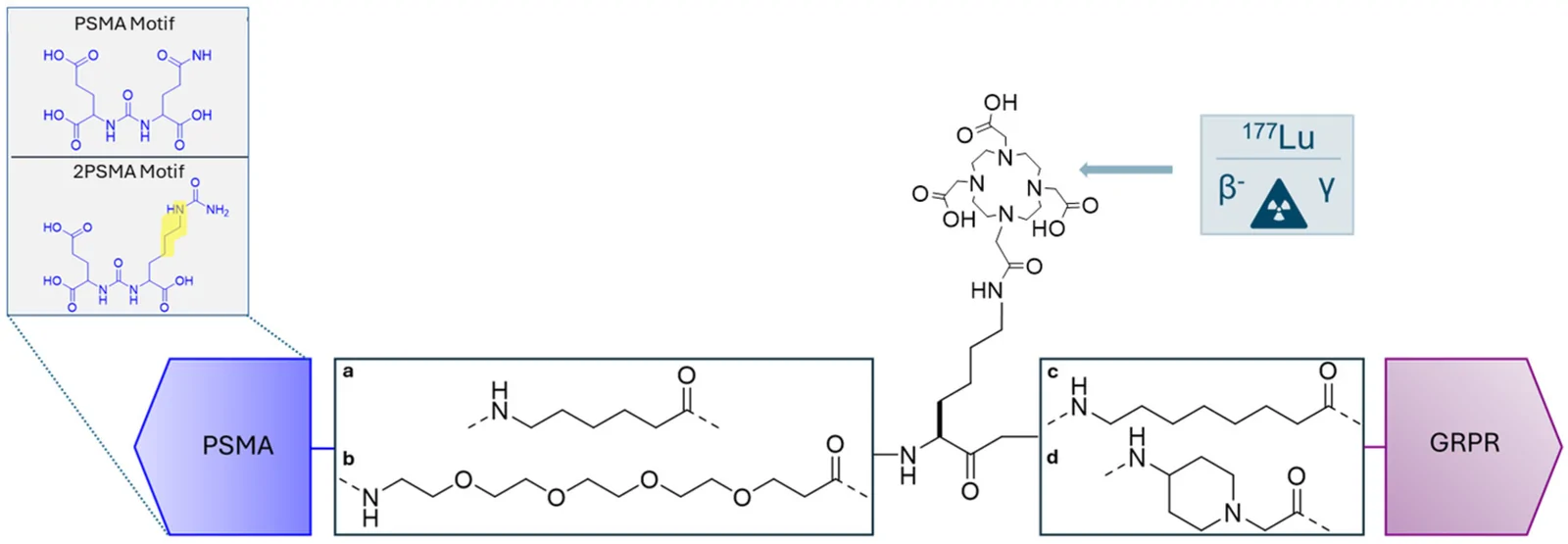
Basic schematic of PSMA/GRPR-targeting heterodimers showing linker variants and core targeting motif modification comparisons; (a) 6AHX, (b) PEG_4_, (c) 8AOC, and (d) CP linkers. The core heterodimer structure is conjugated to DOTA and incorporates either the PSMA motif (**top**) or the 2PSMA motif (**bottom**).

In parallel, for this study, the original DUPA-based motif commonly used in preparation of PSMA inhibitors was modified using a glutamate-ureidolysine (GUL)-like pharmacophore, yielding a new class of compounds denoted “2PSMA” [31,32,33,34,35]. This scaffold is more structurally similar to that used in PSMA-617 and has demonstrated high PSMA affinity in previous studies [36,37,38]. Accordingly, the binding properties of the 2PSMA-R-BBN constructs were evaluated alongside the original DUPA-based PSMA-R-BBN analogs, to assess how linker composition and the PSMA-targeting scaffold modifications influence overall performance. The studies described herein expand upon our previous work with PSMA-6AHX-DOTA-8AOC-BBN by Bandari et al. [23]. The agents, denoted PSMA-6AHX-DOTA-CP-BBN, 2PSMA-6AHX-DOTA-8AOC-BBN, and 2PSMA-PEG_4_-DOTA-8AOC-BBN, are evaluated for radiochemical stability, as well as in vitro and in vivo performance in relevant preclinical animal models (Figure 1). These studies aim to guide rational linker and scaffold design for improved dual-targeting performance in PSMA- and/or GRPR-positive malignancies.

In this work, we report the design and evaluation of a series of PSMA/GRPR-targeting heterodimers conjugated to DOTA to coordinate the clinically established theranostic radionuclide lutetium-177. ([^177^Lu^3+^]Lu)-1,4,7,10-tetraazacyclododecane-1,4,7,10-tetraacetic acid (DOTA)-based radiopharmaceutical agents have often been referred to as the “gold standard” in metal-based radiotherapeutics for a variety of cancers. Advantages of [^177^Lu]Lu-DOTA conjugates include their favorable coordination chemistry, emission profile, and well-established clinical use [39,40,41,42]. [^177^Lu]Lu possesses a β^−^ emission suitable for therapeutic applications, as well as a γ-emission suitable for diagnostic imaging by single-photon emission computed tomography (SPECT). Thus, simultaneous treatment and staging of disease can occur clinically.

## 2. Materials and Methods

### 2.1. General

Preselected GRPR/PSMA-targeting agents, PSMA-6AHX-DOTA-8AOC-BBN, PSMA-6AHX-DOTA-CP-BBN, 2PSMA-6AHX-DOTA-8AOC-BBN, and 2PSMA-PEG_4_-DOTA-8AOC-BBN, were prepared by standard solid-phase peptide synthesis at the University of Missouri (Columbia, MO, USA) at ≥95% purity and used without further purification.

Blocking agents bombesin(1–14) and 2-(Phosphonomethyl)pentanedioic acid were purchased from Sigma-Aldrich Chemical Company (St. Louis, MO, USA) and Tocris Bioscience (Bristol, UK), respectively. [^125^I]I-bombesin was purchased from Revvity (Waltham, MA, USA). [^177^Lu]LuCl_3_ was provided by the University of Missouri Research Reactor (Columbia, MO, USA) in 925 MBq quantities. PSMA-617 was purchased from Selleck Chemicals LLC (Houston, TX, USA). Radiolabeling of [^177^Lu]Lu-PSMA-617 was performed at 90 °C (0.4 M ammonium acetate, pH 5, 37 mBq/nmol, 30 min) and purified via a Shimadzu SCL-10A reversed phase-high performance liquid chromatography (RP-HPLC) system (Kyoto, Japan) with an analytical Phenomenex Jupiter Proteo C-18 column (250 × 4.60 mm, 5 µm) before being placed in DMEM at 100,000 cpm/100 µL.

The PC3 and LNCaP human prostate carcinoma cell lines were purchased from the American Type Tissue Culture Collection (ATCC, Rockland, MD, USA), and the cells were maintained in 45% RPMI 1640, 45% Ham’s F-12, and 10% heat-inactivated fetal bovine serum (FBS). The PC3-PIP cell line was a gift to our research group from Professor Martin Pomper, Johns Hopkins University, which was maintained under the same conditions. GRPR recombinant rabbit monoclonal antibody (18H31L38), F(ab’)_2_-goat anti-rabbit IgG (H + L) secondary antibody conjugated to Alexa Fluor 647 dye, and PSMA monoclonal antibody (GCP-05) conjugated to phycoerythrin (PE) dye were purchased from Thermo Fisher Scientific (Waltham, MA, USA). All other solvents and reagents were purchased from Fisher Scientific (Pittsburgh, PA, USA) or Sigma-Aldrich Chemical Company (St. Louis, MO, USA).

All animal procedures were conducted in accordance with the National Institutes of Health Guide for the Care and Use of Laboratory Animals (8th edition) and were approved by the Institutional Animal Care and Use Committee (IACUC) at the University of Missouri (IACUC #66504) and by the Harry S. Truman Memorial Veterans’ Hospital Subcommittee for Animal Safety (ACORP #292).

### 2.2. Characterization

Liquid chromatography-mass spectrometry (LC-MS) was used to characterize non-metalated heterodimers and the corresponding [^nat^Lu]Lu-complexed heterodimers. All analyses were performed at the University of Missouri Molecular Imaging and Theranostics Center (Columbia, MO, USA). Metalation was conducted in a 1.5:1 molar ratio of natural lutecium chloride to DOTA-conjugated heterodimer (100 µL 0.4 M NH_4_OAc, 100 µL 0.05 M HCl, pH 5.0–6.0) at 80 °C for 45 min.

The 3 min LC-MS gradient consisted of 5% organic phase of acetonitrile and 95% aqueous phase of 0.1% formic acid in water for 0.00–0.50 min, followed by a decrease in the aqueous phase to 5% between 0.51 and 2.00 min. The gradient was held at 5% for 2.00–2.50 min before returning to 95% for 2.51–3.00 min using an Agilent (Santa Clara, CA, USA) C-18 column (2.1 × 50 mm, 2.7 µm).

In addition, reverse phase high-performance liquid chromatography (RP-HPLC) analysis was used for radiometallated compounds via a Shimadzu SCL-10A system (Kyoto, Japan) utilizing a Phenomenex Jupiter Proteo C-18 column (250 × 4.60 mm, 5 µm). The gradient consisted of solvent A (water + 0.1% trifluoroacetic acid) and solvent B (Acetonitrile + 0.1% trifluoroacetic acid) starting at 5% B from 0.0 to 5.0 min, increasing to 95% between 5.0 and 20.0 min, maintaining at 95% for 20.0–25.0, and decreasing to 5% between 25.0 and 25.1 min, and maintaining at 5% until the end of the 30 min gradient.

### 2.3. Flow Cytometry

Flow cytometry was performed on the PC3-PIP (GRPR[+]/PSMA[+]), PC3 (GRPR[+]/PSMA[−]), and LNCaP(GRPR[−]/PSMA[+]) human cancer cell lines. Individually, cells were resuspended in FACS buffer at 1–2 × 10^7^ cells/mL, aliquoted into microcentrifuge tubes, and pelleted by centrifugation. PSMA monoclonal antibodies conjugated to a PE dye and GRPR monoclonal antibodies were separately diluted in FACS buffer. A total of 50 µL of each diluted antibody solution was added separately to each cell suspension. Cells were then incubated at 4 °C for 45 min in the dark. Cells were subssequently washed twice with FACS buffer, with supernatant removal after each wash. To the GRPR stained antibodies, the diluted secondary F(ab’)_2_ goat anti-rabbit antibody conjugated to AlexaFluor 647 dye was added and incubated at 4 °C for 20 min in the dark, followed by two additional washes. Cells were resuspended in 200–500 µL of FACS buffer. Flow cytometry data were acquired as soon as possible after staining, using a Cytek Aurora (Fremont, CA, USA) spectral flow cytometer and analyzed with FlowJo software (Ashland, OR, USA). Fluorescence intensity distributions were normalized to mode for histogram comparison. Cells were first identified on a forward scatter (FSC) versus side scatter (SSC) plot based on their characteristic scatter distribution. Debris and events exhibiting low FSC and high SSC, consistent with dead cells exhibiting reduced cell size and increased intracellular complexity, were excluded. Fluorescence intensity was analyzed within the tightly clustered main cell population in the center of the scatter plot, which was considered viable-cell gate. The GRPR-positive gate was defined using a secondary-antibody-only staining control, whereas the PSMA-positive gate was defined using unstained cells. Fluorescence-intensity distributions were normalized to the mode to account for differences in cell numbers and were displayed as overlaid histograms for comparison.

### 2.4. In Vitro: Competitive Binding Assays

#### 2.4.1. PSMA[+] Cells

LNCaP whole cells were prepared in suspension (7.50 × 10^5^ cells/mL) in binding media (DMEM + 0.2% BSA + 25 mM HEPES). In a microcentrifuge tube, [^177^Lu]LuPSMA-617 was combined at a 1:1:1 ratio with serial dilutions of the bivalent targeting agent (1.5 × 10^3^–2.0 × 10^−4^ nM) and LNCaP cells in binding media. Samples were incubated at room temperature for 60 min, centrifuged, and the supernatant was removed. Cell pellets were washed twice with ice-cold PBS and subsequently counted using an automated gamma counter. An FDA-approved reference compound, PSMA-617, was evaluated in the same manner to confirm assay performance under the experimental conditions used in this study. IC_50_ values were determined by nonlinear regression in GraphPad Prism, with all experiments performed in triplicate.

#### 2.4.2. GRPR[+] Cells

PC3 whole cells were grown in a 24-well attached plate (125,000 cells/well) in growth medium (RPMI, 35 mL NIH FBS, 5 mL L-glutamine, 0.6 mL gentamycin) and incubated for 24 h. The medium was removed, and the cells were washed once with buffer (1X PBS + 0.2% BSA). Aliquots 100 µL of cell binding media (500 mL DMEM + 0.2% BSA + 25 mM HEPES) were added, after which a nM range (1.5 × 10^−4^–2.0 × 10^−4^ nM) of compound, suspended in cell binding media and 100 µL of 100,000 cpm per well of [^125^I]I-bombesin, was added. Cells were incubated at room temperature for 90 min before the total volume in each well was aspirated off. Cells were washed twice with buffer before the cells were lysed by the addition of 2 N NaOH. Cells were subsequently counted in an automated gamma counter. IC_50_ values of the radiotracers were determined by nonlinear regression using GraphPad Prism and completed in triplicate.

### 2.5. Radiolabeling

Radiolabeling of all heterodimer derivatives was conducted by dissolving 1 nmol of each peptide in 100 µL of 0.4 M ammonium acetate and incubating with 470 µCi (17.39 MBq) of [^177^Lu]LuCl_3_ at 80 °C for 45 min (200 µL of 0.05 M HCl, 500 µg of gentisic acid, pH = 5). After incubation, 50 µL of 10 mM DTPA was added to complex unbound radiometal. Radiochemical purity was assessed by RP-HPLC, yielding [^177^Lu]Lu-labeled PSMA-6AHX-[^177^Lu]Lu-DOTA-8AOC-BBN, PSMA-6AHX-[^177^Lu]Lu-DOTA-CP-BBN, 2PSMA-6AHX-[^177^Lu]Lu-DOTA-8AOC-BBN, and 2PSMA-PEG_4_-[^177^Lu]Lu-DOTA-8AOC-BBN with ≥95% radiochemical purity. The radioconjugates were subsequently purified by C-18 Sep-Pak solid-phase extraction prior to all other investigations described herein.

### 2.6. Lipophilicity

Lipophilicity of each radiotracer was determined using the micro-shake-flask method with a 1:1 ratio of 1X PBS (pH 7.4) and n-octanol. After the addition of the radiotracer (~100,000 cpm), the solution was vortexed for 15 min before using a centrifuge to separate the buffer and organic layers. The associated radioactivity in both solvents was measured in a Capintec well counter and used to calculate logD_7.4_.

### 2.7. Stability

Determining stability of all [^177^Lu]Lu-DOTA radiotracer derivatives was completed in 1X phosphate-buffered saline (PBS) and human serum (HS), followed by RP-HPLC analyses at 1, 4, and 24 h. PBS studies were incubated with 100 µL of purified [^177^Lu]Lu-labeled compound in 300 µL of 1X PBS at room temperature (25 °C). For serum stability, 300 µL of human serum was incubated with 100 µL of purified [^177^Lu]Lu-labeled compound at 37 °C. At selected time points, 600 µL of acetonitrile was added to denature the protein before placing in a centrifuge for 5 min at 5000 rpm to form a protein pellet. The supernatant was removed to assess stability via RP-HPLC.

### 2.8. Biodistribution

The biodistribution studies of the new conjugates were completed in male CB-17 SCID mice (n = 4, 4–5 weeks old), which were xenografted with either PC3 or PC3-PIP tumors. Mice were manually randomized into blocks of four to achieve balanced group sizes across designated biodistribution time points. For the ex vivo biodistribution studies, mice were injected intravenously (i.v.) via the tail vein with solid-phase extraction (SPE) purified [^177^Lu]Lu-labeled PSMA-6AHX-DOTA-CP-BBN, 2PSMA-6AHX-DOTA-8AOC-BBN, and 2PSMA-PEG_4_-DOTA-8AOC-BBN at a 470 µCi/nmol (17.4 MBq/nmol) prior to purification. Mice were administered 10–15 µCi (0.37–0.56 MBq) of purified radiolabeled peptide at 0.02–0.03 nmol/mouse in ≤150 µL of isotonic saline. For receptor blocking studies, mice bearing PC3 xenografted tumors were injected with 100 μg of commercially available bombesin(1–14) in isotonic saline 15 min before the radiolabeled compound. Mice bearing PC3-PIP tumors received 2-(phosphonomethyl)-pentanedioic acid (PMPA) in isotonic saline following the same procedure. The mice were euthanized at 1, 4, and 24 h, at which time the tissues and organs were subsequently weighed and counted on a Perkin Elmer Wizard 3 automatic gamma counter, and the percent injected dose per gram (%ID/g) of tissue was calculated by normalizing the percent injected dose to the mass of the tissue in grams.

### 2.9. Imaging

Small animal SPECT/CT imaging was performed on CB-17 SCID mice bearing PC3 and PC3-PIP xenografted tumors at 4 h post-injection of 250 µCi (9.25 MBq) of SPE purified [^177^Lu]Lu-labeled PSMA-6AHX-DOTA-8AOC-BBN, 2PSMA-6AHX-DOTA-8AOC-BBN, or 2PSMA-PEG_4_-DOTA-8AOC-BBN, corresponding to 0.53 nmol/mouse. The starting molar activity prior to purification was 470 µCi/nmol (17.4 MBq/nmol). Due to the low photon abundance of [^177^Lu]Lu, extended SPECT acquisition times were used to enhance image quality and improve visualization of radiotracer distribution. Mice were sacrificed at the 4 h time point prior to imaging. Imaging was then conducted over an 8 h SPECT scan with an additional 5 min CT acquisition. Data was acquired using a small animal MILabs VECTor6CTUHROI (Houten, The Netherlands) SPECT/CT system and reconstructed with MILabs Reconstruction 13.11 (Houten, The Netherlands) for qualitative assessment and correlation with biodistribution data (Photopeak: 208 keV (photopeak window > min 187.2 keV and max 228.8 keV); Energy windows: Emin (keV) = 0 Emax (keV) = 1200; Collimator type: HE-UHR-M 0.75 mm pinhole (High Energy-Ultra High Resolution-Mouse, tungsten cylindrical collimator); Reconstruction algorithm: SROSEM (Similarity-Regulated Ordered Subsets Expectation Maximization) with 0.4 mm voxel size, 128 subsets, 4 iterations; Post filter: Gaussian blurring of 0.6 FWHM (mm). Maximum intensity projection images were displayed using a generalized high-to-low color scale to visualize the overall distribution and areas of radiotracer uptake within each animal. A preliminary live imaging study was also performed using a higher dose (1 mCi/ 37 MBq) over a 30 min SPECT scan with an additional 5 min CT in a live animal maintained on a heating coil with a continuous flow of oxygen with 2.5% isoflurane on the imaging platform.

## 3. Results and Discussion

This study demonstrates successful evaluation of DOTA-conjugated GRPR/PSMA-targeting heterodimers, radiolabeled with the established theranostic radionuclide [^177^Lu]Lu. The work described herein builds upon earlier studies by Bandari et al. that explored a novel dual-targeting approach for prostate cancer imaging and therapy [23,24].

### 3.1. Characterization

LC-MS analysis confirmed successful metal complexation and verified the expected molecular masses of both non-metalated and natural lutetium-complexed heterodimers (Table 1).

### 3.2. Flow Cytometry Analysis

**Figure 2:**
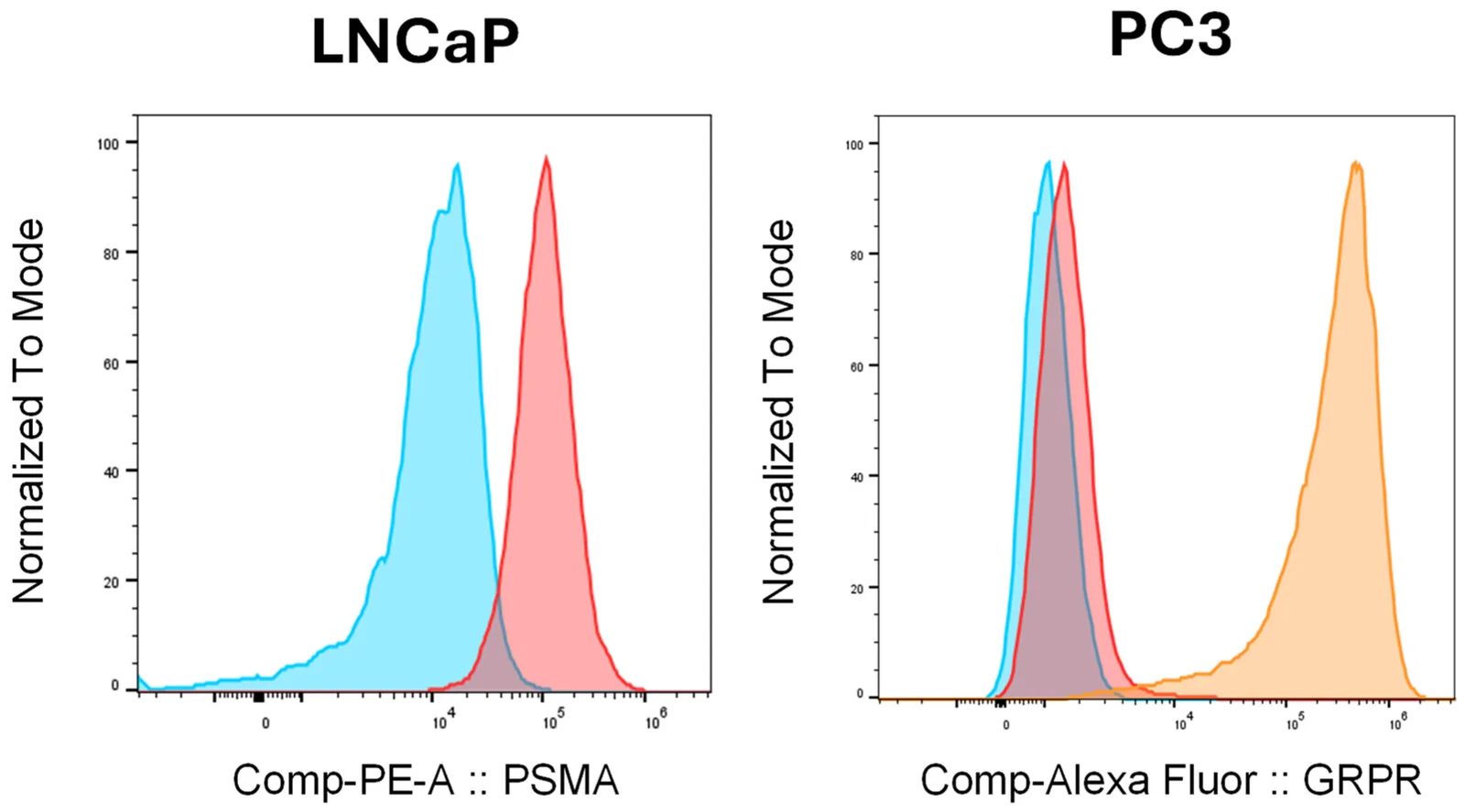
Flow cytometry histograms confirming PSMA expression in LNCaP cells and GRPR expression in PC3 cells. LNCaP cells stained with a PE-conjugated anti-PSMA antibody (red) and PC3 cells stained with an Alexa Fluor-conjugated anti-GRPR antibody (orange) exhibit complete rightward shifts relative to unstained controls (blue). The PE-conjugated anti-PSMA antibody (red) served as a secondary negative control in PC3 cells and showed no rightward shift.

Flow cytometry analysis was performed in LNCaP, PC3, and PC3-PIP cells to confirm target receptor expression levels and validate the use of these cell lines for binding affinity studies in vivo (Figure 2). Histograms represent flow cytometry data normalized to mode to account for differences in total event counts between samples. The highest frequency in each histogram was set to 100% to allow direct comparison of fluorescence intensity distributions.

PSMA expression was assessed by staining LNCaP cells with a PE-conjugated anti-PSMA antibody. Unstained cells (blue; 6600 events) served as the negative control. The stained sample (red; 7301 events) demonstrated a full rightward shift compared to the control, indicating that PSMA is uniformly expressed in this cell line. GRPR expression was evaluated in PC3 cells, where cells were stained with an anti-GRPR primary antibody followed by a fluorescently labeled (Alexa Fluor) secondary antibody. Two negative controls were used including unstained cells (blue; 17,449 events) and secondary antibody-only controls (red; 16,546 events) to assess background and nonspecific binding. A rightward shift in the Alexa Fluor-stained sample (orange; 16,869 events) relative to both controls indicated positive GRPR expression. The complete shift in the histogram suggests uniform GRPR expression across the cell population.

**Figure 3:**
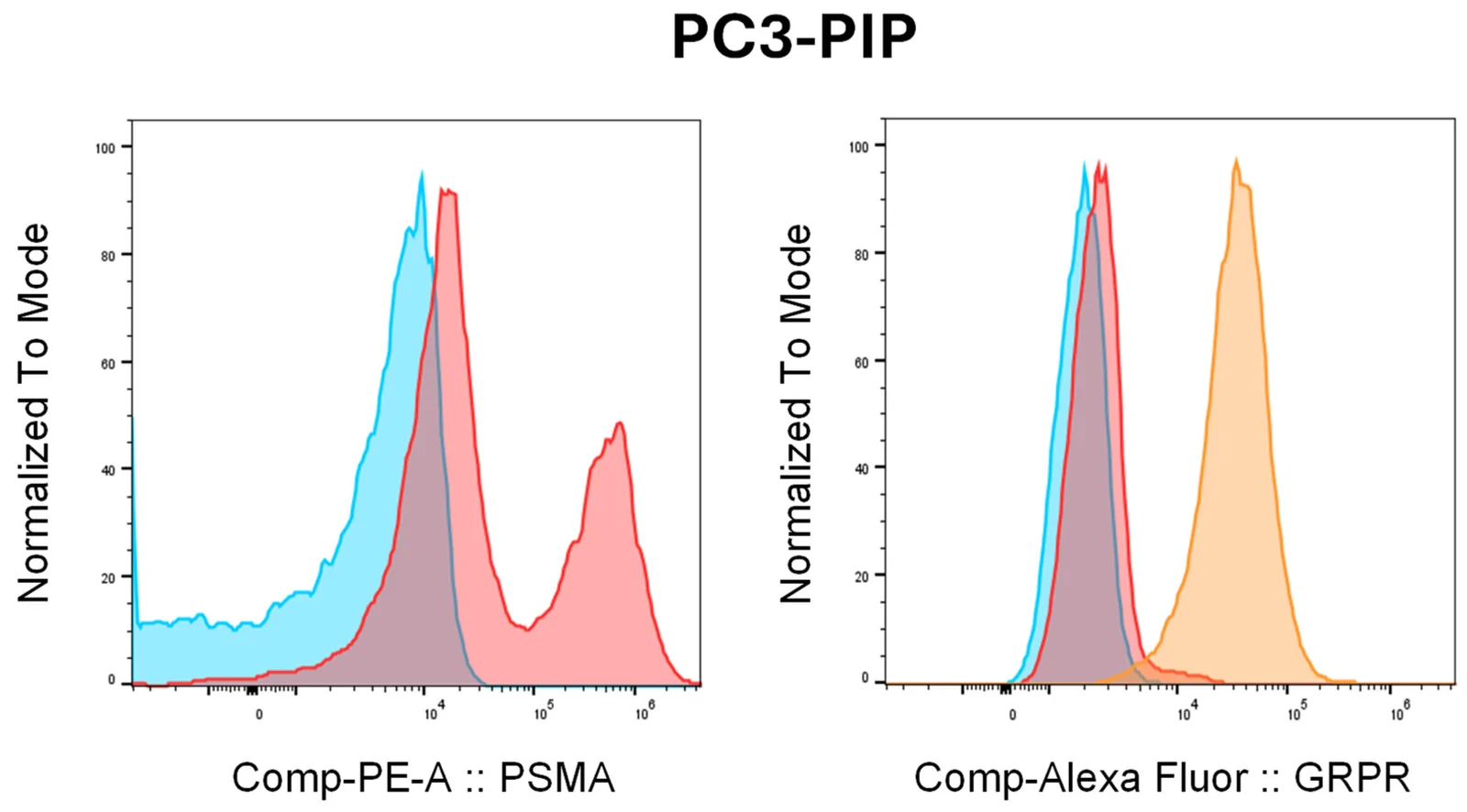
PC3-PIP histograms show background controls (blue-unstained, secondary antibody only) and stained samples, normalized to mode. GRPR staining (orange) demonstrates a uniform rightward shift, indicating widespread expression. PSMA staining demonstrates a bimodal distribution, reflecting partial expression.

Due to the challenge of growing palpable LNCaP tumors on SCID mice, a PC3-PIP tumor model was chosen for assessment of PSMA specificity in vivo [43]. PC3-PIP cells have been reported to express an average of 4.9 × 10^6^ PSMA binding sites/cell; however, they also co-express GRPR [44]. To validate PSMA expression on this cell line, flow cytometry assessment was also performed. The PC3-PIP cells were assessed for expression of both PSMA and GRPR (Figure 3). For PSMA staining, unstained cells (blue; 16,378 events) served as the negative control. The stained sample (red; 14,380 events) exhibits a bimodal distribution, with one peak overlapping the control and a second right-shifted peak, indicating PSMA expression on a subset of cells. For GRPR staining, unstained cells (blue; 16,378 events) and secondary antibody-only controls (red; 17,868 events) were included to assess background and nonspecific binding. A rightward shift in the stained sample (orange; 16,571 events) relative to both controls indicates positive GRPR expression. The complete shift in the histogram suggests that GRPR is uniformly expressed across the cell population.

**Figure 4:**
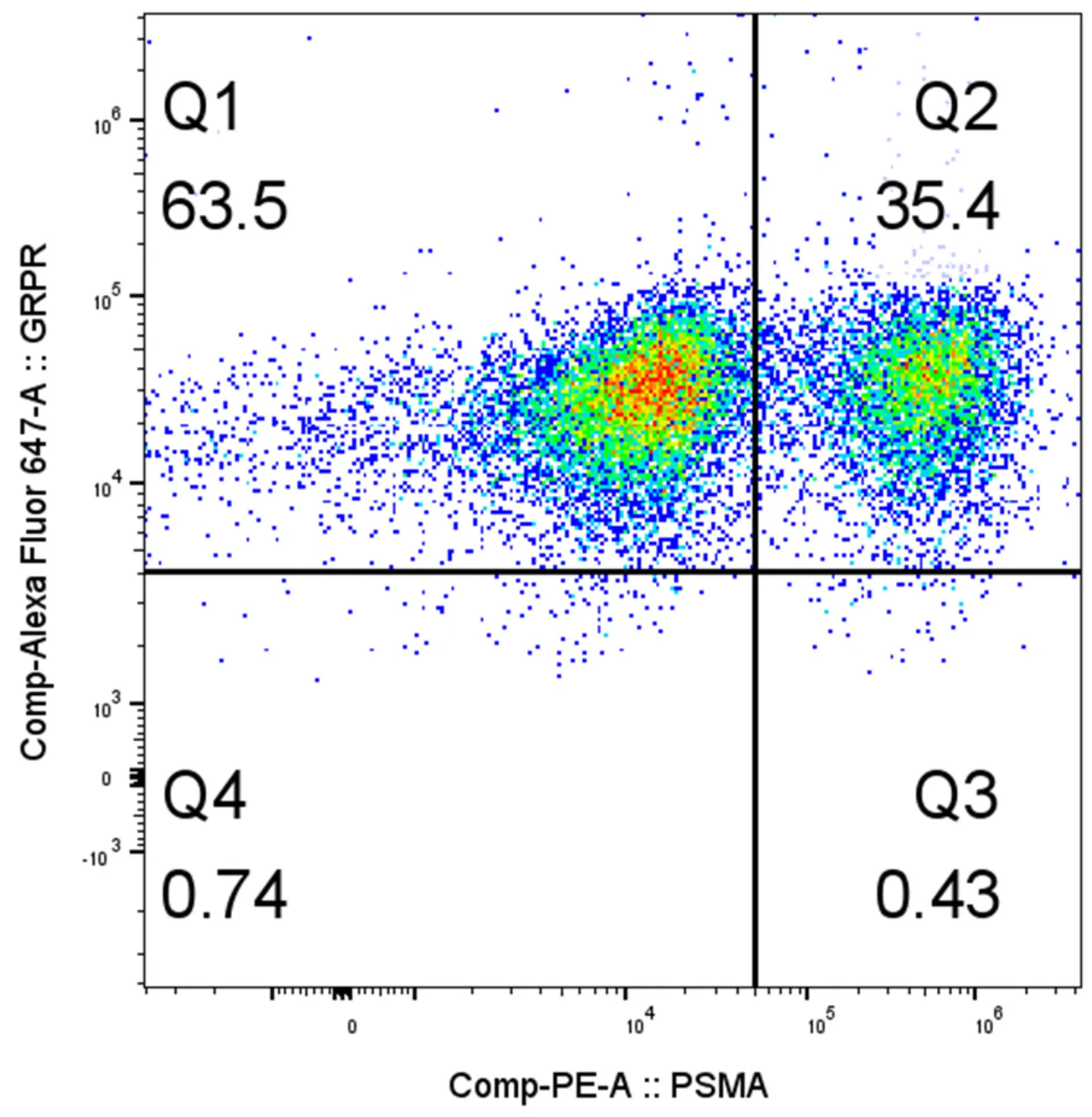
Two-dimensional density plot of live PC3-PIP cells stained for GRPR (y-axis) and PSMA (x-axis). Color intensity reflects cell density. Co-expression analysis shows 35.4% of cells express both receptors and 63.5% express GRPR only.

Co-expression of the PC3-PIP cell line was evaluated using a two-dimensional density plot, with each dot representing a single cell and color intensity corresponding to cell density (Figure 4). The x-axis represents PSMA fluorescence intensity, and the y-axis represents GRPR fluorescence intensity. Quadrant analysis showed that 35.4% of cells co-expressed both GRPR and PSMA, 63.5% of cells expressed only GRPR, and 0.43% expressed PSMA alone. These results confirm that GRPR is broadly expressed across the population, while PSMA expression is limited to a subset of cells.

### 3.3. In Vitro: Competitive Binding Assays

#### PSMA[+] and GRPR[+] Cells

Despite the use of homogenized cell membranes for the enzymatic NAALADase assay to determine binding affinity in our group’s previously published work, ref. [23] the in vivo binding affinities of each heterobivalent conjugate were evaluated using a whole cell competitive displacement assay in LNCaP cells. Whole cell models better preserve native cell-surface receptor expression, receptor conformation, and receptor-mediated interactions compared with membrane-based assays, providing a more physiologically relevant assessment of receptor engagement [45,46,47,48,49].

To ensure this whole cell validation model was appropriately translatable for competitive binding assay assessment, PSMA-617 was tested in triplicate as a reference compound for experimental validation and exhibited an IC_50_ value of 2.7 ± 0.1 nM, consistent with previously reported literature values [50,51,52]. Knowing such, the heterodimers listed in Table 1 were investigated in the same manner to determine how modifications in pharmacokinetic modifier (linker) structure and the PSMA-targeting scaffold influence binding affinity and selectivity. These ligands inhibited the binding of [^177^Lu]Lu-PSMA-617 (Pluvicto^®^) to LNCaP cells in a dose-dependent manner, with experiments performed in triplicate to calculate the IC_50_ values.

**Figure 5:**
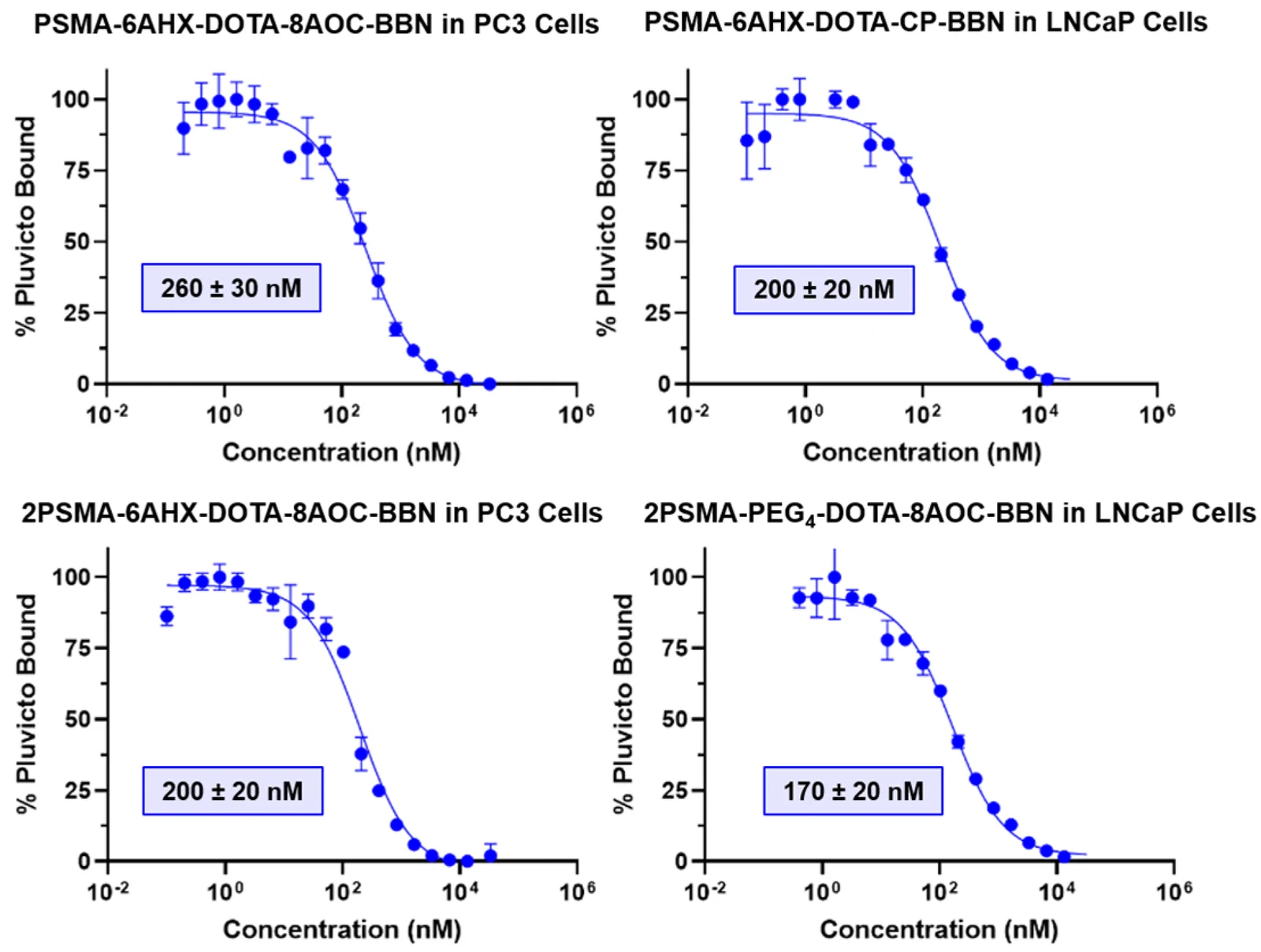
Competitive binding curves of DOTA conjugated PSMA/GRPR-targeting heterobivalent ligands in PSMA[+] LNCaP cells. IC_50_ values were obtained from triplicate assays using [[^177^Lu]Lu-PSMA-617]; all curve fits had R^2^ > 0.98.

PSMA-6AHX-DOTA-8AOC-BBN, PSMA-6AHX-DOTA-CP-BBN, 2PSMA-6AHX-DOTA-8AOC-BBN, and 2PSMA-PEG_4_-DOTA-8AOC-BBN showed only moderate specific binding to the PSMA with IC_50_ values determined to be 260 ± 30, 200 ± 20, 200 ± 20 and, 170 ± 20 nM, respectively (Figure 5).

**Figure 6:**
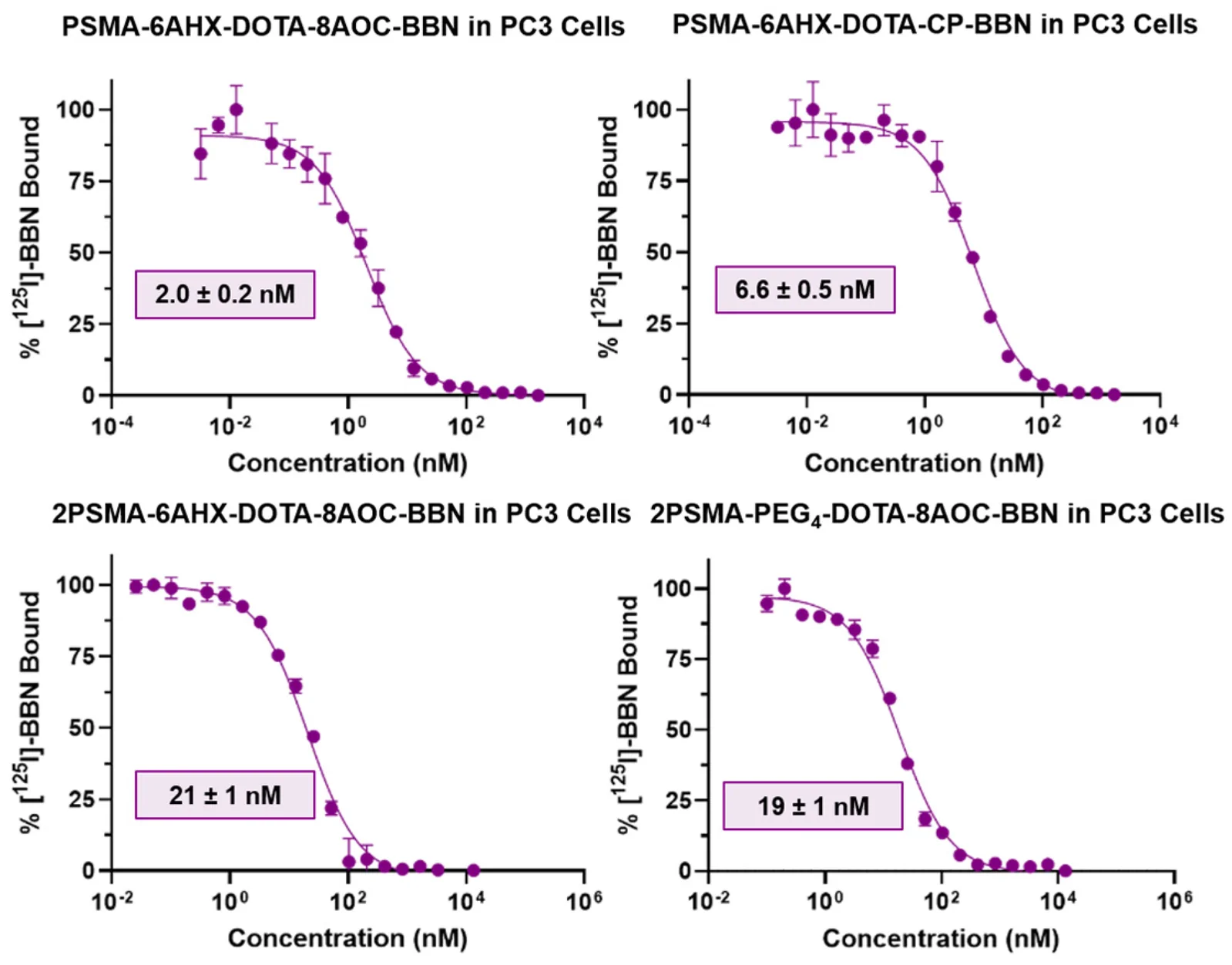
Competitive binding curves of DOTA conjugated PSMA/GRPR-targeting heterobivalent ligands in GRPR[+] PC3 cells. IC_50_ values were obtained from triplicate assays using [[^125^I]I-BBN]; all curve fits had R^2^ > 0.97.

To further characterize the dual-targeting capability of these heterobivalent ligands, their binding affinities to GRPR were assessed in parallel using a cell-based competition assay with PC3 cells (Figure 6). These ligands competed with [^125^I]I-BBN for binding to PC3 cells in a dose-dependent manner. The IC_50_ values for PSMA-6AHX-DOTA-8AOC-BBN, PSMA-6AHX-DOTA-CP-BBN, 2PSMA-6AHX-DOTA-8AOC-BBN, and 2PSMA-PEG_4_-DOTA-8AOC-BBN were calculated in triplicate as 2.0 ± 0.2, 6.6 ± 0.5, 21 ± 1 and, 19 ± 1 nM, respectively.

The resulting IC_50_ values in LNCaP cells to the PSMA-constructs incorporating the more rigid CP linker demonstrated modestly improved PSMA binding in LNCaP cells over the 8AOC-linked analog, but reduced GRPR affinity in PC3 cells. Substitution of the DUPA motif (PSMA) with a GUL-derived 2PSMA scaffold for improved binding produced slight improvements in PSMA binding while generally reducing GRPR affinity, indicating the challenge of maintaining comparable affinity for both receptors. Replacement of the 6AHX spacer with PEG_4_ within the 2PSMA construct yielded modest improvements in PSMA binding with minimal effects on GRPR affinity. Full comparison of the binding affinities in LNCaP and PC3 is shown in Table 2.

While in vitro binding affinity is often used as an indicator of targeting potential, there are reports in the literature that in vitro binding affinity does not always directly translate to in vivo tumor uptake [53,54,55]. For example, the Rosenström group developed two dual-targeting GRPR/PSMA- heterodimers with IC_50_ values of 0.41 ± 0.06 and 4 ± 1 nM for the GRPR targeting motif and high IC_50_ values of 160 ± 30 to 824 ± 230 nM for the PSMA targeting motif [56]. Despite suboptimal IC_50_ values, [^68^Ga]Ga PET and [^111^In]In SPECT tumor-bearing mouse scans resulted in high contrast images with high activity tumor uptake at 1 h p.i. In parallel, a dual-targeting GRPR/α_v_β_3_ agent with moderate IC_50_ values of ~350 nM when evaluated in α_v_β_3_[+] U87-MG glioblastoma cells and ~6 nM for GRPR[+] PC3 cells [57]. Nonetheless, microPET/CT images resulted in discernable tumor uptake with minimal background retention in non-target tissues. With this in mind, in vivo and ex vivo biologic activity was further investigated for GRPR/PSMA- targeting agents.

### 3.4. Radiolabeling

PSMA-6AHX-DOTA-8AOC-BBN, PSMA-6AHX-DOTA-CP-BBN, 2PSMA-6AHX-DOTA-8AOC-BBN, and 2PSMA-PEG_4_-DOTA-8AOC-BBN were each radiolabeled with [^177^Lu]LuCl_3_ (470 µCi/nmol, ≥95%) with very high radiochemical yield and purity.

### 3.5. Physiochemical Characterization

#### 3.5.1. Lipophilicity

The impact of linker variation on radiotracer hydrophilicity and biodistribution has been well reported in the literature [58,59,60]. Lipophilicity investigations (n = 4) at biological pH resulted in LogD_7.4_ values ranging from −2.72 ± 0.03 to −3.3 ± 0.2, deeming all radiotracers to have hydrophilic character.

#### 3.5.2. Stability Assessment

**Figure 7:**
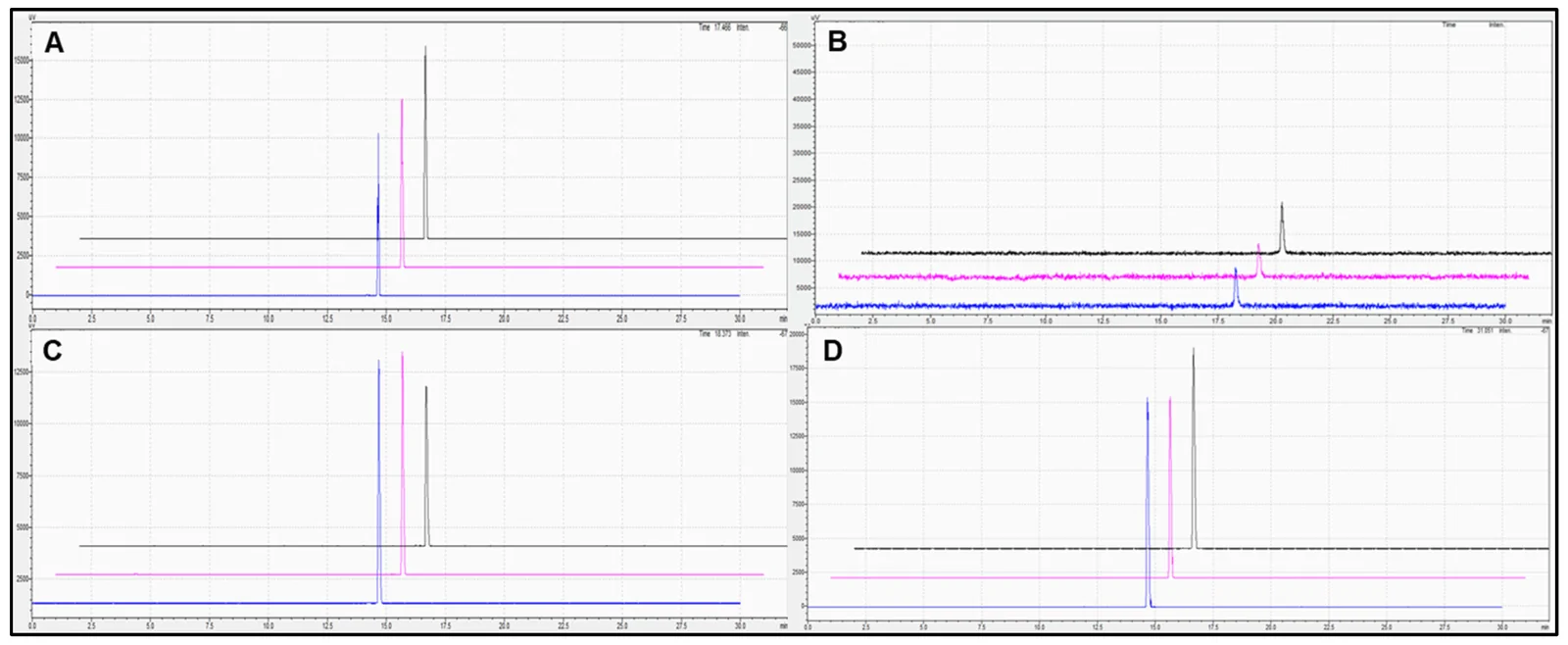
PBS stability studies for (**A**) PSMA-6AHX-[^177^Lu]Lu-DOTA-8AOC-BBN, (**B**) PSMA-6AHX-[^177^Lu]Lu-DOTA-CP-BBN, (**C**) 2PSMA-6AHX-[^177^Lu]Lu-DOTA-8AOC-BBN, and (**D**) 2PSMA-PEG_4_-[^177^Lu]Lu-DOTA-8AOC-BBN RT radiotracers at 1 (blue), 4 (pink), and 24 h (black).

The stability of all [^177^Lu]Lu-DOTA conjugated heterodimers was assessed via RP-HPLC. PBS stability was assessed at 1, 4, and 24 h time points. RP-HPLC analysis demonstrated ≥ 95% intact radioconjugate following 24 h incubation in PBS, with little to no free metal displacement by DTPA (Figure 7).

**Figure 8:**
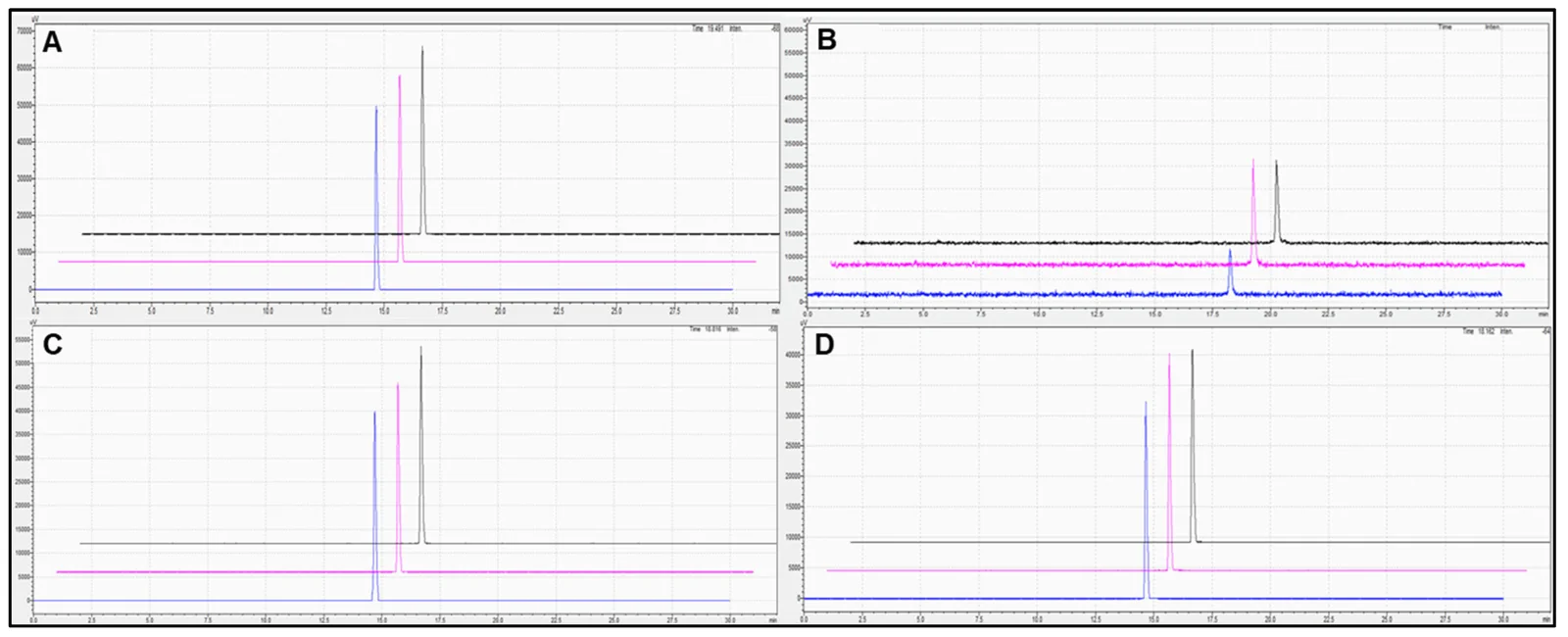
Human serum stability studies for (**A**) PSMA-6AHX-[^177^Lu]Lu-DOTA-8AOC-BBN, (**B**) PSMA-6AHX-[^177^Lu]Lu-DOTA-CP-BBN, (**C**) 2PSMA-6AHX-[^177^Lu]Lu-DOTA-8AOC-BBN, and (**D**) 2PSMA-PEG_4_-[^177^Lu]Lu-DOTA-8AOC-BBN RT radiotracers at 1 (blue), 4 (pink), and 24 h (black).

Subsequent HS stability of each [^177^Lu]Lu-radiolabeled heterodimer was evaluated to determine suitability for in vivo investigations up to 24 h. RP-HPLC analysis demonstrated ≥ 95% intact radioconjugate following 24 h incubation in HS, with ≤5% protein-associated radioconjugate and little to no metal displacement by DTPA, supporting their suitability for further biologic evaluation (Figure 8).

### 3.6. Ex Vivo: Biodistribution Studies

**Figure 9:**
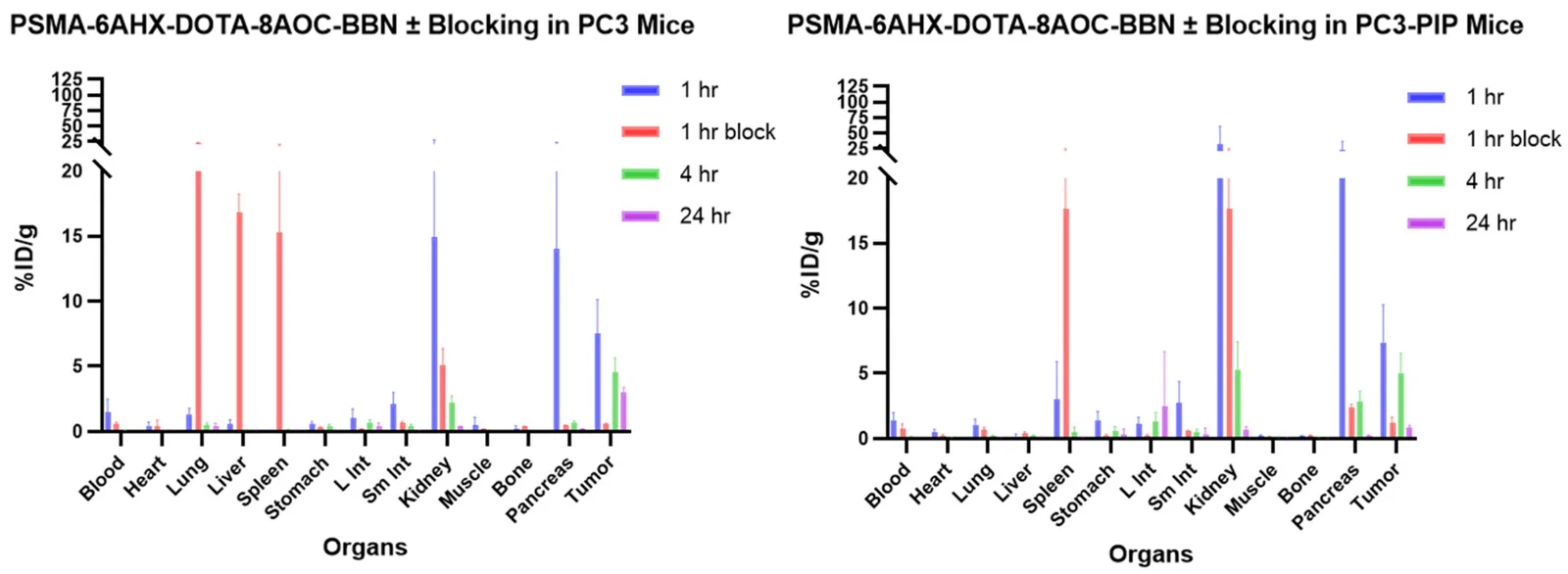
Biodistribution studies of [^177^Lu]Lu-radiolabeled PSMA-6AHX-DOTA-8AOC-BBN in PC3 and PC3-PIP tumor-bearing mice at 1, 4, 4 h block, and 24 h post-intravenous injection using previously reported data from Bandari et al. data for comparison with the current study [23]. Biodistribution results are presented as %ID/g of tissue.

In 2021, Bandari et al. demonstrated measurable uptake of PSMA-6AHX-[^177^Lu]Lu-DOTA-8AOC-BBN in both GRPR- and PSMA-expressing PC3 and PC3-PIP tumor-bearing mice, with rapid renal clearance and favorable pharmacokinetic properties [23]. These previous investigations (Figure 9) show tumor accumulation at 4 h of 4.57 ± 1.07%ID/g in PC3 and 5.04 ± 1.50%ID/g in PC3-PIP xenografted tumors [23]. In the previously described blocking investigations, uptake of the radiotracer was high in normal lung, liver, kidney, and spleen for the PC3 mouse model and high in spleen for PC3-PIP, which is not fully understood but assumed to be associated with retention in blood in these organs. Despite this phenomenon, effective blocking was observed at 1 h p.i. in PC3 (uptake, 7.51 ± 2.61%ID/g; block, 0.58 ± 0.07%ID/g) and PC3-PIP (uptake, 7.31 ± 2.89%ID/g; block, 1.19 ± 0.47%ID/g) xenografted tumors. In the present work, newly generated flow cytometry verified PC3-PIP tumors co-express GRPR (63.5%) and PSMA (35.3%). However, in this 2021 study, the PSMA inhibitor 2-PMPA was the only administered blocking agent prior to the radioconjugate, thus complete blocking was not observed. A more complete blocking assay is needed, which would include a cocktail of 2-PMPA and BBN 1–14 and could be used to delineate the contribution of each targeting moiety for either GRPR or PSMA.

In the current work, ex vivo biodistribution studies were performed to evaluate radiotracer uptake across major organs and assess the effects of incremental modifications to the heterodimer scaffold based on the previously published data from our lab. The pharmacokinetic properties of each [^177^Lu]Lu-radiolabeled heterodimer were observed after intravenous (i.v.) injection in both PC3 and PC3-PIP tumor-bearing mice at 1, 4, and 24 h. A more complete assessment to evaluate the therapeutic potential of these new agents would require biodistribution analyses at extended time points. Blocking studies were also conducted at 4 h p.i. to assess the specificity of the novel cell-targeting agents for tumor tissue. In addition, accumulation of the radiotracer in the pancreas, an organ known to express the GRPR in very high numbers, refs. [61,62,63] was moderate to high for all tracers in PC3 and PC3-PIP tumor-bearing mice at 1 h, with minimal retention at the 24 h time point. All radiotracers were observed to be predominately excreted via the renal-urinary pathway after the 1 h time point. Notably, moderate uptake in the kidney was observed across all studies, which was also observed during small animal SPECT/CT imaging investigations.

**Figure 10:**
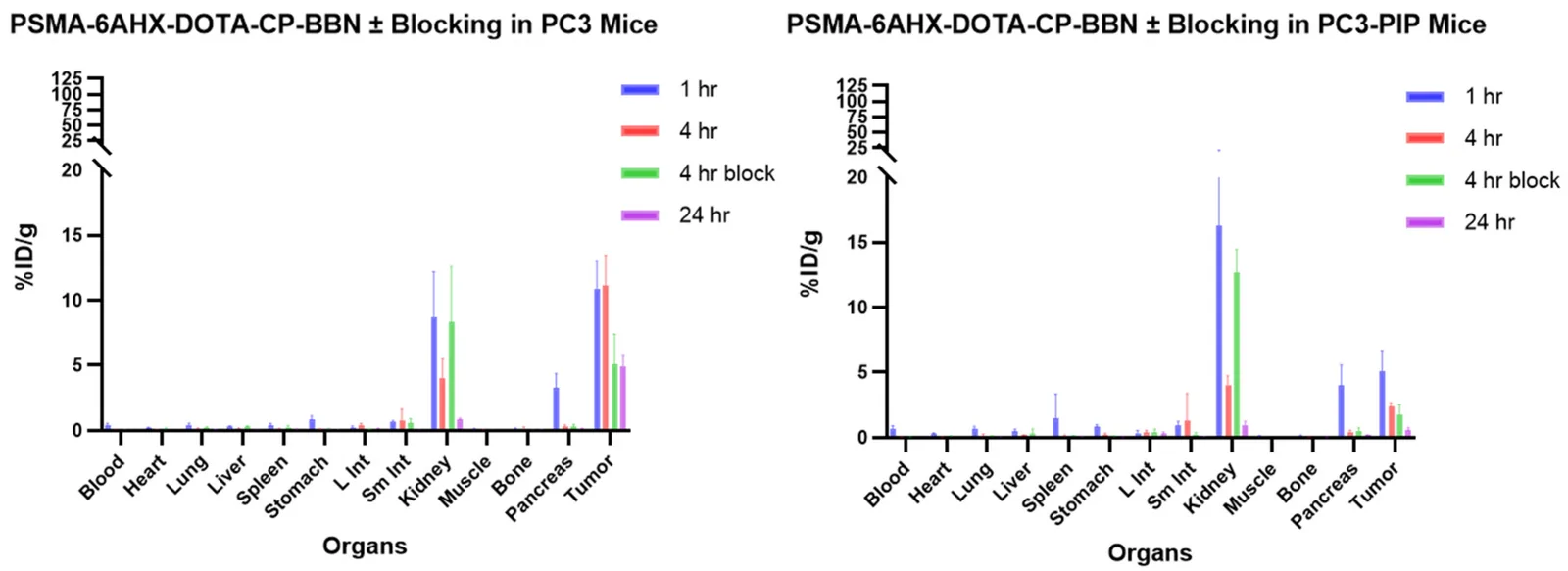
Biodistribution studies of [^177^Lu] labeled PSMA-6AHX-DOTA-CP-BBN in PC3 and PC3-PIP tumor-bearing mice at 1, 4, 4 h block, and 24 h post intravenous injection. Biodistribution results are presented as %ID/g of tissue.

Among the heterodimers evaluated in this study, PSMA-6AHX-[^177^Lu]Lu-DOTA-CP-BBN was first assessed for biodistribution in PC3 and PC3-PIP tumor-bearing mice, demonstrating a tumor uptake of 11.14 ± 2.35 and 2.36 ± 0.31%ID/g at 4 h p.i., respectively (Figure 10). Blocking studies at 4 h demonstrated an uptake of 5.05 ± 2.35%ID/g in PC3 and 1.75 ± 0.79%ID/g in PC3-PIP tumors, which showed partially effective blocking of the receptor. While favorable uptake was observed in GRPR[+] tumor models, accumulation in PSMA[+] models was limited. This variation in the heterodimer is hypothesized to exhibit excessive rigidity which may impact binding to cell-surface receptors. This property is attributed to the structural constraints imposed by the CP linker attached to the GRPR-targeting moiety. This low tumor uptake in PC3-PIP models ultimately precluded further in vivo investigations of the PSMA-6AHX-DOTA-CP-BBN heterodimer.

**Figure 11:**
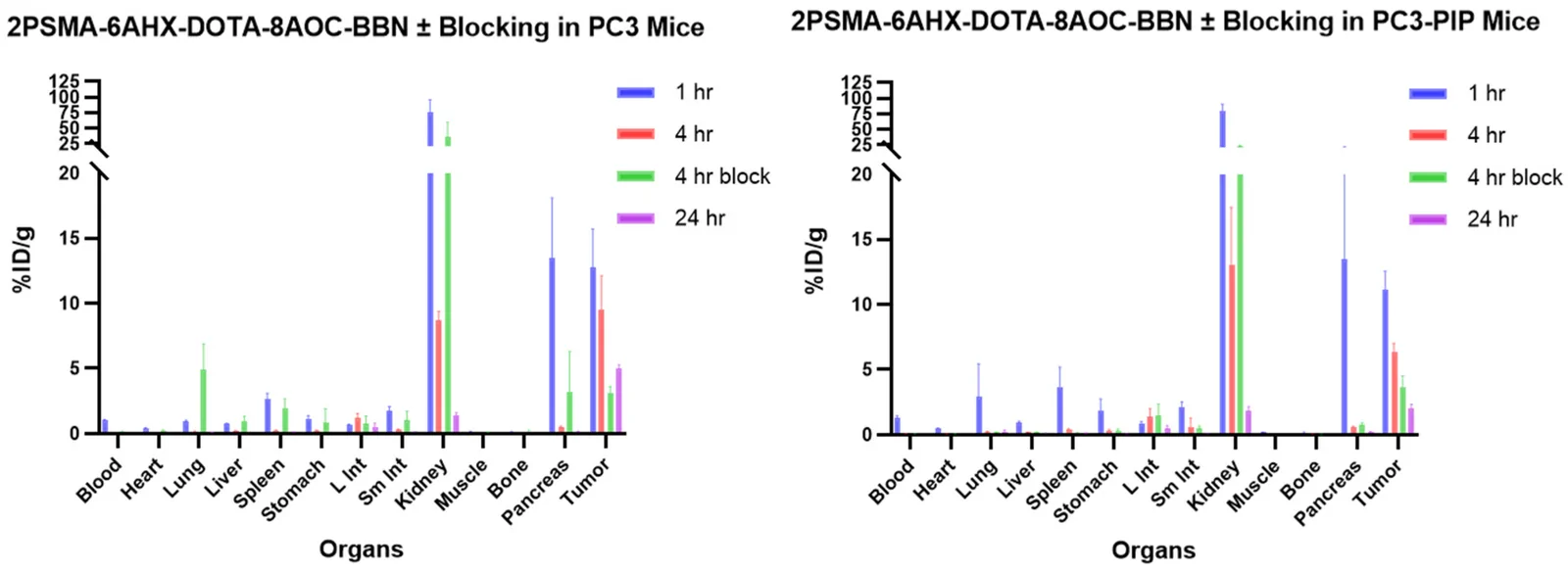
Biodistribution studies of [^177^Lu] labeled 2PSMA-6AHX-DOTA-8AOC-BBN in PC3 and PC3-PIP tumor-bearing mice at 1, 4, 4 h block, and 24 h post intravenous injection. Biodistribution results are presented as %ID/g of tissue.

Biodistribution studies for 2PSMA-6AHX-[^177^Lu]Lu-DOTA-8AOC-BBN in PC3 and PC3-PIP tumor-bearing mice demonstrated tumor uptake values at 4 h of 9.52 ± 2.59%ID/g and 6.37 ± 0.64%ID/g, respectively (Figure 11). Effective blocking was demonstrated at 4 h with a reduced uptake of 3.12 ± 0.50%ID/g in PC3 and 3.63 ± 0.89%ID/g in PC3-PIP tumors. In comparison to PSMA-6AHX-[^177^Lu]Lu-DOTA-8AOC-BBN, which contains the first iteration of the DUPA PSMA-targeting moiety, the adjusted GUL PSMA-targeting moiety of 2PSMA-6AHX-[^177^Lu]Lu-DOTA-8AOC-BBN modestly increased tumor uptake in PSMA[+] PC3-PIP tumors.

**Figure 12:**
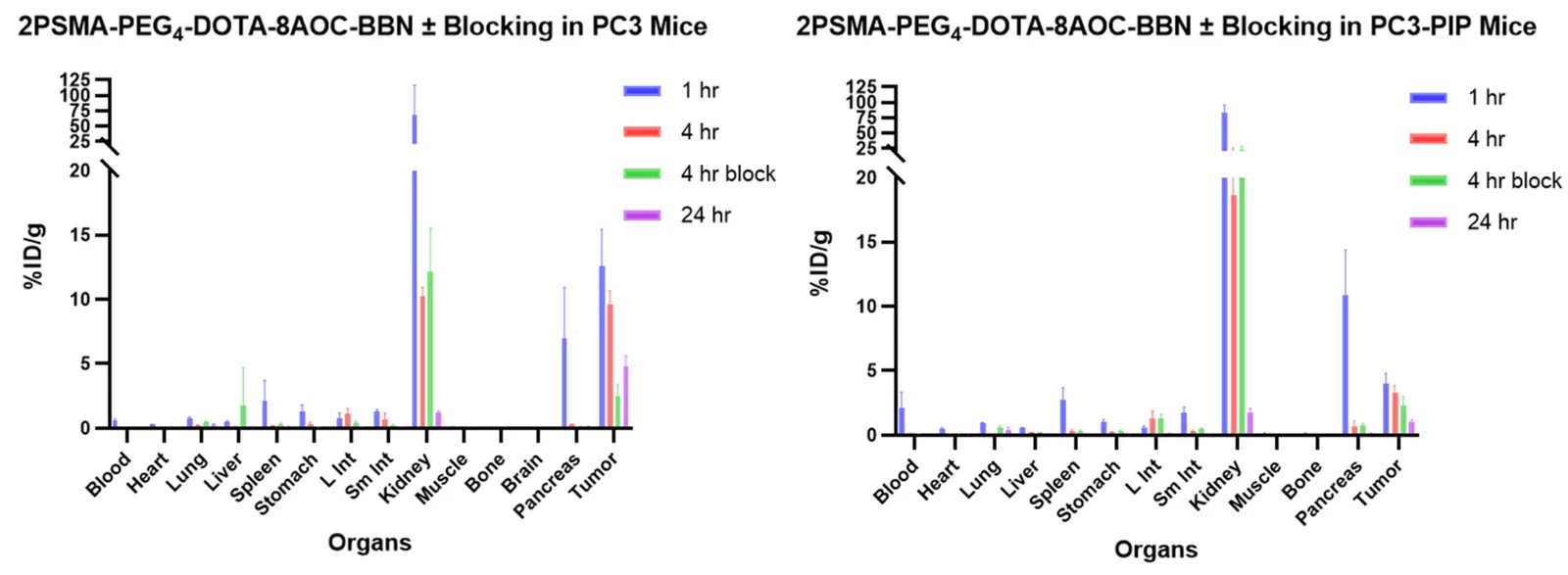
Biodistribution studies of [^177^Lu]Lu-radiolabeled 2PSMA-PEG4-DOTA-8AOC-BBN in PC3 and PC3-PIP tumor-bearing mice at 1, 4, 4 h block, and 24 h post intravenous injection. Biodistribution results are presented as %ID/g of tissue.

Biodistribution investigations for 2PSMA-PEG_4_-[^177^Lu]Lu-DOTA-8AOC-BBN in PC3 and PC3-PIP tumor-bearing mice demonstrated tumor accumulations at 4 h p.i. of 9.63 ± 1.03 and 3.30 ± 0.55%ID/g, respectively (Figure 12). The reduced tumor uptake observed in PSMA[+] models may be attributed to the hydrophilic PEG_4_ linker on the PSMA-targeting motif, which likely interacts unfavorably with the hydrophobic binding pocket of PSMA [64]. Although the increased flexibility of the PEG_4_ linker was hypothesized to reduce steric hindrance and enhance receptor binding, the overall hydrophilicity of the construct may have instead promoted rapid clearance from the tumor site [65,66]. At the 1 h time point, tumor uptake was higher, with values of 12.54 ± 2.90%ID/g in PC3 tumors and 4.03 ± 0.75%ID/g in PC3-PIP tumors, supporting the notion that this increased hydrophilicity resulted in reduced tumor retention due to rapid clearance from the blood [67]. Notably, kidney accumulation was relatively high at 1 h p.i. (PC3, 69.11 ± 47.47; PC3-PIP, 83.67 ± 12.02%ID/g) and at 4 h p.i. (PC3, 10.22 ± 0.69; PC3-PIP, 18.63 ± 6.17%ID/g), indicating slower renal clearance in comparison to all other heterodimers tested.

The reduced PSMA receptor prevalence determined by the flow cytometric analysis (PSMA 35.4%; GRPR 63.5%) may partially contribute to lower tumor uptake in the PC3-PIP xenografted mouse model. The biodistribution studies demonstrated that PSMA-6AHX-DOTA-CP-BBN is 26% PSMA mediated, 2PSMA-6AHX-[^177^Lu]Lu-DOTA-8AOC-BBN is 43% mediated, and 2PSMA-PEG_4_-[^177^Lu]Lu-DOTA-8AOC-BBN is 31% PSMA mediated. Furthermore, 2PSMA-6AHX-[^177^Lu]Lu-DOTA-8AOC-BBN exhibited a higher tumor uptake 4 h p.i. than compounds PSMA-6AHX-DOTA-CP-BBN and 2PSMA-PEG_4_-[^177^Lu]Lu-DOTA-8AOC-BBN. Thus, this data indicates that with low PSMA expression in PC3-PIP cell lines, heterobivalent agents require strong PSMA mediation to improve binding. These findings also suggest that additional factors, including receptor density, tracer internalization, and compound-specific pharmacokinetic properties, also influence tumor accumulation. One limitation of the present study is the number of animals evaluated at each biodistribution time point (n = 4), which may limit the ability to detect subtle differences between groups. Future studies incorporating larger cohorts may provide additional statistical resolution of these differences.

To further evaluate ex vivo contrast, tumor:organ ratios (tumor:muscle, tumor:blood, tumor:kidney, tumor:liver, and tumor:pancreas) were evaluated at 1, 4, and 24 h p.i. to assess tracer specificity, nontarget clearance, and retention over time in both PC3-PIP and PC3 tumor-bearing mice. Favorable tumor:blood and tumor:muscle ratios were observed, indicating rapid systemic clearance and minimal/nominal nonspecific uptake in benign tissues. As anticipated for PSMA-targeting radioligands, high renal activity was observed which was demonstrated in low tumor:kidney ratios. Similarly, due to physiologic GRPR expression, low tumor:pancreas ratios were observed. In PC3-PIP tumor bearing mouse models, all tumor:organ values are consistently lower across all time points in comparison to PC3 tumor bearing models. This may reflect a combination of rapid radiotracer clearance from the tumor and increased nonspecific uptake of the radiopharmaceutical in non-target tissues. For tumor:blood and tumor:kidney ratios, all values increased over time (with the exception of PSMA-6AHX-[^177^Lu]Lu-DOTA-CP-BBN in tumor:blood), indicating effective clearance from blood and the renal system, while maintaining tumor retention. The observed trend in tumor:muscle and tumor:liver ratios were highest at 4 h p.i. with a decrease in all heterodimer values out to 24 h p.i. Overall, each radiotracer exhibits relatively high tumor:organ ratios, and these findings support the tracer’s specificity and sustained retention in PSMA[+] and GRPR[+] tumors over time (Table 3 and Table 4).

### 3.7. In Vivo: SPECT/CT Imaging

**Figure 13:**
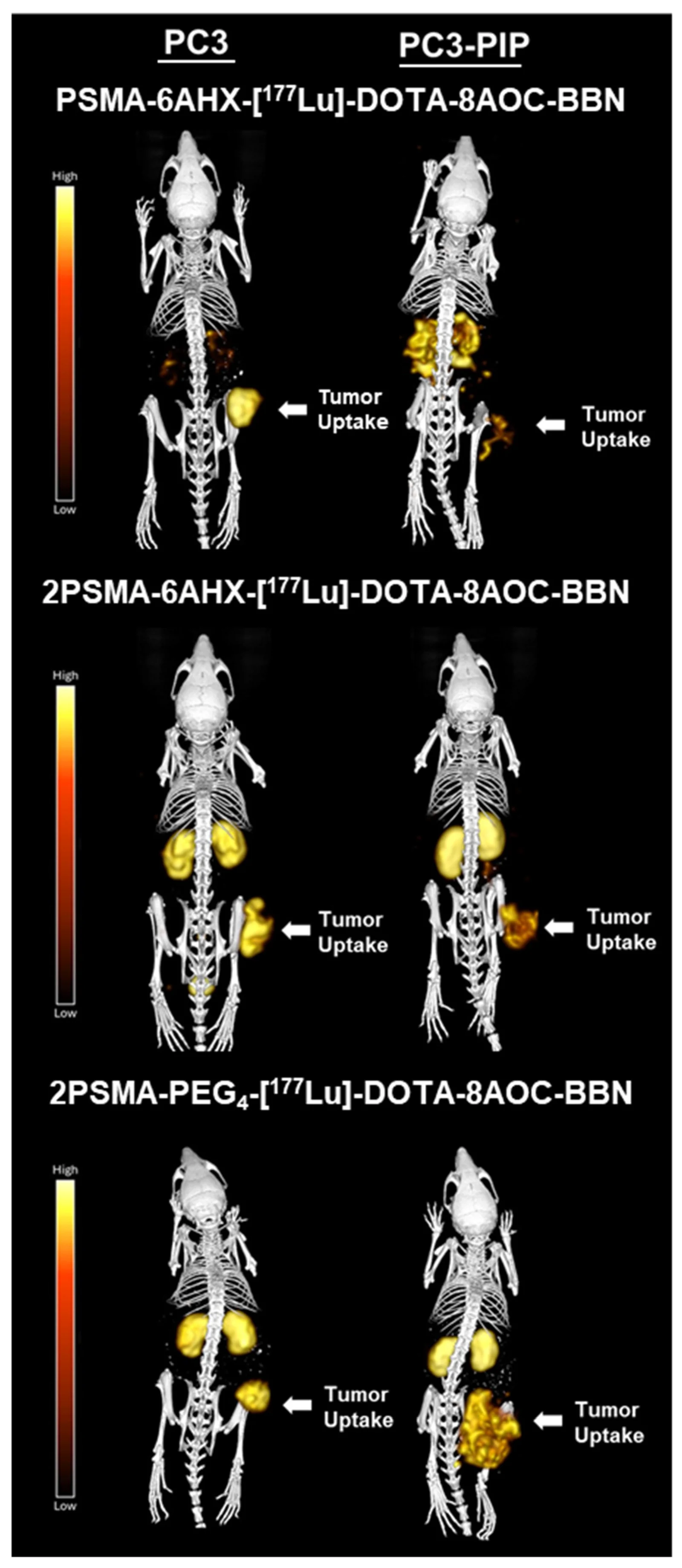
Whole-body SPECT/CT images of PSMA-6AHX-[^177^Lu]Lu-, 2PSMA-6AHX-[^177^Lu]Lu-DOTA-8AOC-BBN, and 2PSMA-PEG_4_-[^177^Lu]Lu-DOTA-8AOC-BBN in PC3 and PC3-PIP tumor-bearing mice at 4 h p.i.

Overall, the biodistribution profile supported subsequent investigation of small animal SPECT/CT imaging, utilizing the gamma emissions of [^177^Lu]Lu. Thus, small animal SPECT/CT imaging was performed to assess in vivo tracer distribution and retention in PC3 and PC3-PIP tumor-bearing mice at 4 h p.i. Images produced were qualitative, with clear and selective tumor localization in both PSMA and GRPR expressing tumors (Figure 13), alongside distinct kidney localization that was observed across all SPECT/CT scans. This finding is consistent with the expression of PSMA in renal tissue and the kidney’s role as a major excretory organ [68,69]. Incorporation of the 2PSMA motif resulted in distinct PSMA[+] tumor visualization in imaging investigations and modestly enhanced tumor uptake observed in biodistribution experiments. Despite this modification also increased kidney retention as observed by ex vivo analysis, the heterodimer 2PSMA-6AHX-[^177^Lu]Lu-DOTA-8AOC-BBN demonstrated promise as a dual-targeting theranostic agent for PCa, with opportunities for further optimization of linker design to improve biodistribution and tumor detection. These qualitative imaging observations further support the quantitative ex vivo biodistribution studies, confirming the tracer’s specificity and sustained retention in PSMA[+]/GRPR[+] tumors.

The agents described are compatible for a range of rare-earth and rare-earth-like theranostic radiometals. However, the use of alternative DOTA-based BFCAs to yield kinetically stable and inert radioconjugates for PCa targeting is currently being evaluated. These heterobivalent tracers address key limitations associated with single receptor targeting monovalent probes by enabling simultaneous targeting of both PSMA and GRPR.

## 4. Conclusions

The work described herein evaluates heterobivalent [^177^Lu]Lu-radioconjugates with potential for usage as a theranostic targeting platform for PSMA- and/or GRPR-expressing PCa. The agents evaluated were chosen to examine pharmacokinetic effects ranging from structural modifications involving carboxypiperdine and polyethylene glycol crosslinker chemistry to modifications of the PSMA binding motif (Table 5). They demonstrated favorable tumor targeting and imaging characteristics, with 2PSMA-6AHX-[^177^Lu]Lu-DOTA-8AOC-BBN showing the most promise after careful experimental evaluation. This construct exhibited improved localization in receptor-expressing tumors, with favorable accumulation in tumors and desirable clearance properties from non-target tissues. 2PSMA-6AHX-[^177^Lu]Lu-DOTA-8AOC-BBN demonstrated highly favorable tumor:muscle uptake in comparison to all other constructs evaluated and demonstrated favorable tumor retention at the 4 h time point in both PC3 and PC3-PIP tumor models. These findings support the potential utility of a dual-targeting strategy to address tumor heterogeneity. A limitation of the present study is the absence of direct GRPR/PSMA- targeting controls and individual receptor-specific blocking experiments. This limits definitive assessment of each targeting moiety and the advantages of the heterobivalent approach. While receptor-specific blocking has been previously investigated for PSMA-6AHX-DOTA-8AOC-BBN radiotracer, inclusion of these controls in future studies will further define the contributions of PSMA/GRPR-mediated targeting in current heterodimeric constructs described herein [23]. Additional studies are warranted to evaluate the therapeutic and translational potential of this agent and to optimize pharmacokinetic performance further through minor, systematic modifications to linker and targeting vector design. Exploration of alternative chelator systems and additional theranostic radionuclide pairs, including [^64/67^Cu]Cu, may also provide opportunities to expand the versatility of this molecular design. Ultimately, the design of these agents for targeted cancer treatment could advance personalized medicine approaches in oncology and should continue to be explored.

## Figures and Tables

**Table 1:** Mass spectrometry and LogD_7.4_ values and molecular weight of each DOTA-conjugated heterodimer.

Compound	Calculated Molecular Mass	Observed m/z ^1^	LogD_7.4_ Value ^2^
PSMA-6AHX-DOTA-8AOC-BBN	2357.5	2356.6	−3.2 ± 0.16
PSMA-6AHX-DOTA-CP-BBN	2355.4	2355.8	−3.3 ± 0.24
2PSMA-6AHX-DOTA-8AOC-BBN	2400.1	2399.9	−2.7 ± 0.03
2PSMA-PEG_4_-DOTA-8AOC-BBN	2532.2	2531.6	−3.3 ± 0.23

^1^ Each heterodimer labeled with [^nat^Lu]Cl_3_. ^2^ Each heterodimer labeled with [^177^Lu]Cl_3_.

**Table 2:** Comparative IC_50_ values (nM) of reported PSMA/GRPR-targeting heterodimers determined in LNCaP (PSMA[+]) and PC3 (GRPR[+]) cell lines.

Compound	LNCaP (PSMA[+])	PC3 (GRPR[+])
PSMA-6AHX-DOTA-8AOC-BBN	260 ± 30	2.0 ± 0.2
PSMA-6AHX-DOTA-CP-BBN	200 ± 20	6.6 ± 0.5
2PSMA-6AHX-DOTA-8AOC-BBN	200 ± 20	21 ± 1
2PSMA-PEG_4_-DOTA-8AOC-BBN	170 ± 20	19 ± 1

**Table 3:** Tumor:organ contrast, expressed as uptake ratios calculated from biodistribution data (%ID/g) at 1, 4, 24 h post-injection in PC3-PIP tumor-bearing mice.

Compound	Time Point	Tumor: Muscle	Tumor: Blood	Tumor: Kidney	Tumor: Liver	Tumor: Pancreas
PSMA-6AHX-DOTA-8AOC-BBN *	**1**	45.9 ± 10	5.4 ± 1	0.4 ± 0	10.5 ± 1	0.4 ± 0
	**4**	503.7 ± 362	42.5 ± 18	1.0 ± 0	23.8 ± 4	1.9 ± 1
	**24**	95.8 ± 61	105.8 ± 44	1.3 ± 0	8.7 ± 0.7	7.0 ± 6
PSMA-6AHX-DOTA-CP-BBN	**1**	53.8 ± 18	7.4 ± 2	0.3 ± 0	11.9 ± 4	1.5 ± 1
	**4**	205.6 ± 65	121.3 ± 81	0.6 ± 0	15.1 ± 4	6.3 ± 2
	**24**	107.0 ± 42	107 ± 42	0.6 ± 0	6.5 ± 3	3.3 ± 1
2PSMA-6AHX-DOTA-8AOC-BBN	**1**	73.7 ± 0.5	8.7 ± 0.5	0.1 ± 0	12.21 ± 0.4	3.3 ± 5
	**4**	569.5 ± 199	130.1 ± 60	0.6 ± 0	40.32 ± 7	89.9 ± 156
	**24**	403.5 ± 66	344.3 ± 85	1.1 ± 0	19.7 ± 2	16.6 ± 7
2PSMA-PEG_4_-DOTA-8AOC-BBN	**1**	65.1 ± 19	8.9 ± 2	0.1 ± 0	15.0 ± 4	0.8 ± 0
	**4**	287.1 ± 46	91.3 ± 40	0.4 ± 0	29.1 ± 5	10.3 ± 5
	**24**	602.3 ± 425	194.5 ± 86	0.7 ± 0	16.8 ± 3	15.8 ± 9

* Indicates data analyzed from published Bandari et al. work for comparison with the current study [23].

**Table 4:** Tumor:organ contrast, expressed as uptake ratios calculated from biodistribution data (%ID/g) at 1, 4, 24 h post-injection in PC3 tumor-bearing mice.

Compound	Time Point	Tumor: Muscle	Tumor: Blood	Tumor: Kidney	Tumor: Liver	Tumor: Pancreas
PSMA-6AHX-DOTA-8AOC-BBN *	**1**	53.7 ± 33	6.8 ± 3	0.7 ± 0.3	14.0 ± 5	11.9 ± 15
	**4**	572.8 ± 259	113.8 ± 39	2.2 ± 0.8	37.5 ± 9	7.2 ± 3
	**24**	451.5 ± 176	399.9 ± 306	8.5 ± 2	40.9 ± 13	19.3 ± 6
PSMA-6AHX-DOTA-CP-BBN	**1**	169.9 ± 83	31.1 ± 9	1.4 ± 0.5	45.7 ± 13	3.6 ± 1.2
	**4**	710.6 ± 407	842.8 ± 331	2.9 ± 0.6	75.3 ± 10	37.7 ± 8
	**24**	574.5 ± 416	732.5 ± 287	6.16 ± 1	55 ± 5	32.3 ± 2
2PSMA-6AHX-DOTA-8AOC-BBN	**1**	89.9 ± 27	13.3 ± 4	0.2 ± 0.1	17.6 ± 5	1.1 ± 0
	**4**	872.6 ± 400	299.2 ± 100	1.1 ± 0.2	51.4 ± 16	23.4 ± 10
	**24**	739.8 ± 277	874.0 ± 276	3.6 ± 0.7	42.3 ± 4	42.0 ± 13
2PSMA-PEG_4_-DOTA-8AOC-BBN	**1**	118.7 ± 20	23.9 ± 2	0.2 ± 0	90.3 ± 126	2.3 ± 1.5
	**4**	828.8 ± 206	700.9 ± 204	0.9 ± 0	71.3 ± 2	150.4 ± 241
	**24**	707.3 ± 212	973.0 ± 153	4.1 ± 0	41.6 ± 6	61.8 ± 37

* Indicates data analyzed from published Bandari et al. work for comparison with the current study [23].

**Table 5:** Comparison of receptor binding (IC_50_ values), tumor uptake, kidney uptake, and tumor:muscle ratios of selected PSMA/GRPR-targeting radiopharmaceuticals.

	PSMA				GRPR			
[^177^Lu]Lu-Labeled Compound	IC_50_ (nM)	Tumor Uptake 4 h (%ID/g)	Kidney Uptake 4 h (%ID/g)	Tumor: Muscle 4 h	IC_50_ (nM)	Tumor Uptake 4 h (%ID/g)	Kidney Uptake 4 h (%ID/g)	Tumor: Muscle 4 h
PSMA-6AHX-DOTA-8AOC-BBN	260 ± 30	5.04 ± 1.50 *	5.31 ± 0.21 *	503.7 ± 362 *	2.0 ± 0.2	4.57 ± 1.07 *	2.17 ± 0.57 *	572.8 ± 259 *
PSMA-6AHX-DOTA-CP-BBN	200 ± 20	2.36 ± 0.31	4.05 ± 0.69	205.6 ± 65	6.6 ± 0.5	11.14 ± 2.35	4.00 ± 1.48	710.6 ± 407
2PSMA-6AHX-DOTA-8AOC-BBN	200 ± 20	6.37 ± 0.64	13.03 ± 4.43	569.5 ± 199	21 ± 1	9.52 ± 2.59	8.67 ± 0.70	872.6 ± 400
PSMA-PEG_4_-DOTA-8AOC-BBN	170 ± 20	3.30 ± 0.55	18.63 ± 6.17	287.1 ± 46	19 ± 1	9.63 ± 1.03	10.22 ± 0.69	828.8 ± 206

* Indicates data analyzed from published Bandari et al. work for comparison with the current study [23].

## Data Availability

All of the research generated from these investigations will be made available for public access under NIH Open Access Guidelines and are available at PubMed Central. The data presented in this study are included in the article/Supplementary Materials.

## Supplementary Materials

The following supporting information can be downloaded at: https://www.mdpi.com/article/10.3390/cancers18203252/s1, Supplemental Data.

## Abbreviations

The following abbreviations are used in this manuscript:

PCa	Prostate cancer
PSMA	Prostate-specific membrane antigen
GRPR	Gastrin-releasing peptide receptor
DOTA	1,4,7,10-tetraazacyclododecane-1,4,7,10-tetraacetic acid
SPECT	Single Photon Emission Computed Tomography
8AOC	8-Aminooctanoic acid
CP	Carboxypiperidine
PEG	Polyethylene glycol
6AHX	6-Aminohexanoic acid
LC-MS	Liquid chromatography-mass spectrometry
RP-HPLC	Reversed-phase high performance liquid chromatography
FBS	Fetal bovine serum
2-PMPA	2-(Phosphonomethyl)pentanedioic acid
BBN	Bombesin
HS	Human serum
CT	Computed Tomography
PE	Phycoerythrin

## References

[1] Siegel R.L., Kratzer T.B., Wagle N.S., Sung H., Jemal A. (2026). Cancer statistics, 2026. CA.

[2] Wynant G.E., Murphy G.P., Horoszewicz J.S., Neal C.E., Collier B.D., Mitchell E., Purnell G., Tyson I., Heal A., Abdel-Nabi H. (1991). Immunoscintigraphy of prostatic cancer: Preliminary results with 111In-labeled monoclonal antibody 7E11-C5.3 (CYT-356). Prostate.

[3] Hennrich U., Eder M. (2021). [(68)Ga]Ga-PSMA-11: The First FDA-Approved (68)Ga-Radiopharmaceutical for PET Imaging of Prostate Cancer. Pharmaceuticals.

[4] Afshar-Oromieh A., Hetzheim H., Kübler W., Kratochwil C., Giesel F.L., Hope T.A., Eder M., Eisenhut M., Kopka K., Haberkorn U. (2016). Radiation dosimetry of (68)Ga-PSMA-11 (HBED-CC) and preliminary evaluation of optimal imaging timing. Eur. J. Nucl. Med. Mol. Imaging.

[5] Hennrich U., Eder M. (2022). [(177)Lu]Lu-PSMA-617 (Pluvicto(TM)): The First FDA-Approved Radiotherapeutical for Treatment of Prostate Cancer. Pharmaceuticals.

[6] Bakht M.K., Beltran H. (2025). Biological determinants of PSMA expression, regulation and heterogeneity in prostate cancer. Nat. Rev. Urol..

[7] Chambers C., Chitwood B., Smith C.J., Miao Y. (2024). Elevating theranostics: The emergence and promise of radiopharmaceutical cell-targeting heterodimers in human cancers. iRADIOLOGY.

[8] Wright G.L., Grob B.M., Haley C., Grossman K., Newhall K., Petrylak D., Troyer J., Konchuba A., Schellhammer P.F., Moriarty R. (1996). Upregulation of prostate-specific membrane antigen after androgen-deprivation therapy. Urology.

[9] Emmett L., Crumbaker M., Ho B., Willowson K., Eu P., Ratnayake L., Epstein R., Blanksby A., Horvath L., Guminski A. (2019). Results of a Prospective Phase 2 Pilot Trial of 177Lu–PSMA-617 Therapy for Metastatic Castration-Resistant Prostate Cancer Including Imaging Predictors of Treatment Response and Patterns of Progression. Clin. Genitourin. Cancer.

[10] Jones W., Griffiths K., Barata P.C., Paller C.J. (2020). PSMA theranostics: Review of the current status of PSMA-targeted imaging and radioligand therapy. Cancers.

[11] Ramnaraign B., Sartor O. (2023). PSMA-targeted radiopharmaceuticals in prostate cancer: Current data and new trials. Oncologist.

[12] Verhoeven M., Ruigrok E.A., van Leenders G.J., van den Brink L., Balcioglu H.E., van Weerden W.M., Dalm S.U. (2023). GRPR versus PSMA: Expression profiles during prostate cancer progression demonstrate the added value of GRPR-targeting theranostic approaches. Front. Oncol..

[13] Fernández R., Soza-Ried C., Iagaru A., Stephens A., Müller A., Schieferstein H., Sandoval C., Amaral H., Kramer V. (2023). Imaging GRPr Expression in Metastatic Castration-Resistant Prostate Cancer with [(68)Ga]Ga-RM2-A Head-to-Head Pilot Comparison with [(68)Ga]Ga-PSMA-11. Cancers.

[14] Faviana P., Boldrini L., Erba P., Di Stefano I., Manassero F., Bartoletti R. (2021). Gastrin-releasing peptide receptor in low grade prostate cancer: Can it be a better predictor than prostate-specific membrane antigen?. Front. Oncol..

[15] Baratto L., Song H., Duan H., Hatami N., Bagshaw H.P., Buyyounouski M., Hancock S., Shah S., Srinivas S., Swift P. (2021). PSMA- and GRPR-Targeted PET: Results from 50 Patients with Biochemically Recurrent Prostate Cancer. J. Nucl. Med..

[16] Baratto L., Jadvar H., Iagaru A. (2018). Prostate Cancer Theranostics Targeting Gastrin-Releasing Peptide Receptors. Mol. Imaging Biol..

[17] Liu S. (2009). Radiolabeled Cyclic RGD Peptides as Integrin αvβ3-Targeted Radiotracers: Maximizing Binding Affinity via Bivalency. Bioconjug. Chem..

[18] Fischer G., Schirrmacher R., Waengler B., Wängler C. (2013). Radiolabeled heterobivalent peptidic ligands: An approach with high future potential for in vivo imaging and therapy of malignant diseases. ChemMedChem.

[19] Dijkgraaf I., Kruijtzer J.A., Liu S., Soede A.C., Oyen W.J., Corstens F.H., Liskamp R.M., Boerman O.C. (2007). Improved targeting of the αvβ3 integrin by multimerisation of RGD peptides. Eur. J. Nucl. Med. Mol. Imaging.

[20] Bandari R.P., Jiang Z., Reynolds T.S., Bernskoetter N.E., Szczodroski A.F., Bassuner K.J., Kirkpatrick D.L., Rold T.L., Sieckman G.L., Hoffman T.J. (2014). Synthesis and biological evaluation of copper-64 radiolabeled [DUPA-6-Ahx-(NODAGA)-5-Ava-BBN (7-14) NH2], a novel bivalent targeting vector having affinity for two distinct biomarkers (GRPr/PSMA) of prostate cancer. Nucl. Med. Biol..

[21] Durkan K., Jiang Z., Rold T.L., Sieckman G.L., Hoffman T.J., Bandari R.P., Szczodroski A.F., Liu L., Miao Y., Reynolds T.S. (2014). A heterodimeric [RGD-Glu-[64Cu-NO2A]-6-Ahx-RM2] αvβ3/GRPr-targeting antagonist radiotracer for PET imaging of prostate tumors. Nucl. Med. Biol..

[22] Rivera-Bravo B., Ramírez-Nava G., Mendoza-Figueroa M.J., Ocampo-García B., Ferro-Flores G., Ávila-Rodríguez M.A., Santos-Cuevas C. (2021). [68Ga]Ga-iPSMA-Lys3-Bombesin: Biokinetics, dosimetry and first patient PET/CT imaging. Nucl. Med. Biol..

[23] Bandari R.P., Carmack T.L., Malhotra A., Watkinson L., Fergason Cantrell E.A., Lewis M.R., Smith C.J. (2021). Development of Heterobivalent Theranostic Probes Having High Affinity/Selectivity for the GRPR/PSMA. J. Med. Chem..

[24] Bandari R.P., Lewis M.R., Smith C.J. (2018). Synthesis and Evaluation of [DUPA-6-Ahx-Lys (DOTA)-6-Ahx-RM2], a Novel, Bivalent Targeting Ligand for GRPr/PSMA Biomarkers of Prostate Cancer. Chem. Biol. Lett..

[25] Guo H., Miao Y. (2014). Introduction of an 8-aminooctanoic acid linker enhances uptake of 99mTc-labeled lactam bridge–cyclized α-MSH peptide in melanoma. J. Nucl. Med..

[26] Desantis J., Mammoli A., Eleuteri M., Coletti A., Croci F., Macchiarulo A., Goracci L. (2022). PROTACs bearing piperazine-containing linkers: What effect on their protonation state?. RSC Adv..

[27] Minamimoto R., Sonni I., Hancock S., Vasanawala S., Loening A., Gambhir S.S., Iagaru A. (2018). Prospective evaluation of 68Ga-RM2 PET/MRI in patients with biochemical recurrence of prostate cancer and negative findings on conventional imaging. J. Nucl. Med..

[28] Wieser G., Popp I., Christian Rischke H., Drendel V., Grosu A.-L., Bartholomä M., Weber W.A., Mansi R., Wetterauer U., Schultze-Seemann W. (2017). Diagnosis of recurrent prostate cancer with PET/CT imaging using the gastrin-releasing peptide receptor antagonist 68 Ga-RM2: Preliminary results in patients with negative or inconclusive [18 F] Fluoroethylcholine-PET/CT. Eur. J. Nucl. Med. Mol. Imaging.

[29] Koller L., Joksch M., Schwarzenböck S., Kurth J., Heuschkel M., Holzleitner N., Beck R., Von Amsberg G., Wester H.-J., Krause B.J. (2023). Preclinical Comparison of the ^64^Cu- and ^68^Ga-Labeled GRPR-Targeted Compounds RM2 and AMTG, as Well as First-in-Humans [^68^Ga]Ga-AMTG PET/CT. J. Nucl. Med..

[30] Hamidi M., Azadi A., Rafiei P. (2006). Pharmacokinetic consequences of pegylation. Drug Deliv..

[31] Graham K., Lesche R., Gromov A.V., Böhnke N., Schäfer M., Hassfeld J., Dinkelborg L., Kettschau G. (2012). Radiofluorinated Derivatives of 2-(Phosphonomethyl)pentanedioic Acid as Inhibitors of Prostate Specific Membrane Antigen (PSMA) for the Imaging of Prostate Cancer. J. Med. Chem..

[32] Young J.D., Ma M.T., Eykyn T.R., Atkinson R.A., Abbate V., Cilibrizzi A., Hider R.C., Blower P.J. (2021). Dipeptide inhibitors of the prostate specific membrane antigen (PSMA): A comparison of urea and thiourea derivatives. Bioorg. Med. Chem. Lett..

[33] Roy J., Nguyen T.X., Kanduluru A.K., Venkatesh C., Lv W., Reddy P.V.N., Low P.S., Cushman M. (2015). DUPA Conjugation of a Cytotoxic Indenoisoquinoline Topoisomerase I Inhibitor for Selective Prostate Cancer Cell Targeting. J. Med. Chem..

[34] Kozikowski A.P., Nan F., Conti P., Zhang J., Ramadan E., Bzdega T., Wroblewska B., Neale J.H., Pshenichkin S., Wroblewski J.T. (2001). Design of Remarkably Simple, Yet Potent Urea-Based Inhibitors of Glutamate Carboxypeptidase II (NAALADase). J. Med. Chem..

[35] Kozikowski A.P., Zhang J., Nan F., Petukhov P.A., Grajkowska E., Wroblewski J.T., Yamamoto T., Bzdega T., Wroblewska B., Neale J.H. (2004). Synthesis of urea-based inhibitors as active site probes of glutamate carboxypeptidase II: Efficacy as analgesic agents. J. Med. Chem..

[36] Hoareau R., Bach-Gansmo T., Cumming P., Olberg D.E. (2022). A new automated and putatively versatile synthesis of the PSMA-ligand derivative [18F]DCFPyL using the FASTlabTM synthesizer. EJNMMI Radiopharm. Chem..

[37] Kratochwil C., Giesel F.L., Stefanova M., Benešová M., Bronzel M., Afshar-Oromieh A., Mier W., Eder M., Kopka K., Haberkorn U. (2016). PSMA-Targeted Radionuclide Therapy of Metastatic Castration-Resistant Prostate Cancer with ^177^Lu-Labeled PSMA-617. J. Nucl. Med..

[38] Chen Y., Foss C.A., Byun Y., Nimmagadda S., Pullambhatla M., Fox J.J., Castanares M., Lupold S.E., Babich J.W., Mease R.C. (2008). Radiohalogenated Prostate-Specific Membrane Antigen (PSMA)-Based Ureas as Imaging Agents for Prostate Cancer. J. Med. Chem..

[39] Kaur J., Mukherjee M. (2024). An Overview of Structural Framework and Classification of Theranostic Radiopharmaceuticals. ChemistrySelect.

[40] Chakravarty R., Chakraborty S. (2021). A review of advances in the last decade on targeted cancer therapy using (177)Lu: Focusing on (177)Lu produced by the direct neutron activation route. Am. J. Nucl. Med. Mol. Imaging.

[41] Li L., Jaraquemada-Peláez M.G., Kuo H.T., Merkens H., Choudhary N., Gitschtaler K., Jermilova U., Colpo N., Uribe-Munoz C., Radchenko V. (2019). Functionally Versatile and Highly Stable Chelator for (111)In and (177)Lu: Proof-of-Principle Prostate-Specific Membrane Antigen Targeting. Bioconjug Chem..

[42] Zhu Y., Zhong G., Zhang W., Wei W., Yin B., Zhang K. (2026). Combination therapy strategies in engineered 177Lu radiopharmaceuticals beyond single modality: Design principles and application outlet. Cell Commun. Signal..

[43] Liberio M.S., Sadowski M.C., Soekmadji C., Davis R.A., Nelson C.C. (2014). Differential Effects of Tissue Culture Coating Substrates on Prostate Cancer Cell Adherence, Morphology and Behavior. PLoS ONE.

[44] Kiess A.P., Minn I., Chen Y., Hobbs R., Sgouros G., Mease R.C., Pullambhatla M., Shen C.J., Foss C.A., Pomper M.G. (2015). Auger Radiopharmaceutical Therapy Targeting Prostate-Specific Membrane Antigen. J. Nucl. Med..

[45] Wüstemann T., Bauder-Wüst U., Schäfer M., Eder M., Benesova M., Leotta K., Kratochwil C., Haberkorn U., Kopka K., Mier W. (2016). Design of Internalizing PSMA-specific Glu-ureido-based Radiotherapeuticals. Theranostics.

[46] Tiffany C.W., Slusher B.S. (2002). Measurement of glutamate carboxypeptidase II (NAALADase) enzyme activity by the hydrolysis of [^3^H]-N-acetylaspartylglutamate (NAAG). Curr. Protoc. Pharmacol..

[47] Ullrich M., Brandt F., Löser R., Pietzsch J., Wodtke R. (2023). Comparative Saturation Binding Analysis of (64)Cu-Labeled Somatostatin Analogues Using Cell Homogenates and Intact Cells. ACS Omega.

[48] Motulsky H.J., Mahan L.C., Ansel P.A. (1985). Radioligand, agonists and membrane receptors on intact cells: Data analysis in a bind. Trends Pharmacol. Sci..

[49] Vauquelin G., Van Liefde I., Swinney D.C. (2015). Radioligand binding to intact cells as a tool for extended drug screening in a representative physiological context. Drug Discov. Today Technol..

[50] Tönnesmann R., Meyer P.T., Eder M., Baranski A.C. (2019). [(177)Lu]Lu-PSMA-617 Salivary Gland Uptake Characterized by Quantitative In Vitro Autoradiography. Pharmaceuticals.

[51] Murce E., Beekman S., Spaan E., Handula M., Stuurman D., de Ridder C., Seimbille Y. (2023). Preclinical Evaluation of a PSMA-Targeting Homodimer with an Optimized Linker for Imaging of Prostate Cancer. Molecules.

[52] Hirata S., Mishiro K., Higashi T., Fuchigami T., Munekane M., Arano Y., Kinuya S., Ogawa K. (2022). Synthesis and evaluation of a multifunctional probe with a high affinity for prostate-specific membrane antigen (PSMA) and bone. Nucl. Med. Biol..

[53] Takamura Y., Kakuta H. (2021). In Vivo Receptor Visualization and Evaluation of Receptor Occupancy with Positron Emission Tomography. J. Med. Chem..

[54] Huber H.J., Mistry H.B. (2024). Explaining in-vitro to in-vivo efficacy correlations in oncology pre-clinical development via a semi-mechanistic mathematical model. J. Pharmacokinet. Pharmacodyn..

[55] Sakahara H., Endo K., Koizumi M., Nakashima T., Kunimatsu M., Watanabe Y., Kawamura Y., Nakamura T., Tanaka H., Kotoura Y. (1988). Relationship between in vitro binding activity and in vivo tumor accumulation of radiolabeled monoclonal antibodies. J. Nucl. Med..

[56] Mitran B., Varasteh Z., Abouzayed A., Rinne S.S., Puuvuori E., De Rosa M., Larhed M., Tolmachev V., Orlova A., Rosenström U. (2019). Bispecific GRPR-Antagonistic Anti-PSMA/GRPR Heterodimer for PET and SPECT Diagnostic Imaging of Prostate Cancer. Cancers.

[57] Bandara N., Stott Reynolds T.J., Schehr R., Bandari R.P., Diebolder P.J., Krieger S., Xu J., Miao Y., Rogers B.E., Smith C.J. (2018). Matched-pair, (86)Y/(90)Y-labeled, bivalent RGD/bombesin antagonist, [RGD-Glu-[DO3A]-6-Ahx-RM2], as a potential theranostic agent for prostate cancer. Nucl. Med. Biol..

[58] Anselmi M., Borbély A., Figueras E., Michalek C., Kemker I., Gentilucci L., Sewald N. (2021). Linker Hydrophilicity Modulates the Anticancer Activity of RGD-Cryptophycin Conjugates. Chemistry.

[59] Kwon D., Zhang Z., Zeisler J., Kuo H.T., Lin K.S., Benard F. (2022). Reducing the Kidney Uptake of High Contrast CXCR4 PET Imaging Agents via Linker Modifications. Pharmaceutics.

[60] Guo H., Yang J., Gallazzi F., Miao Y. (2011). Effects of the amino acid linkers on the melanoma-targeting and pharmacokinetic properties of 111In-labeled lactam bridge–cyclized α-MSH peptides. J. Nucl. Med..

[61] Wang L., Chapple D.E., Kuo H.-T., Kurkowska S., Wilson R.P., Lau W.S., Ng P., Uribe C., Bénard F., Lin K.-S. (2025). Novel 177Lu-Labeled [Thz14]Bombesin(6–14) Derivatives with Low Pancreas Accumulation for Targeting Gastrin-Releasing Peptide Receptor-Expressing Cancer. Pharmaceuticals.

[62] Jensen R., Battey J., Spindel E., Benya R. (2008). International Union of Pharmacology. LXVIII. Mammalian bombesin receptors: Nomenclature, distribution, pharmacology, signaling, and functions in normal and disease states. Pharmacol. Rev..

[63] Bakker I.L., Fröberg A.C., Busstra M.B., Verzijlbergen J.F., Konijnenberg M., Van Leenders G.J.L.H., Schoots I.G., De Blois E., Van Weerden W.M., Dalm S.U. (2021). GRPr Antagonist ^68^Ga-SB3 PET/CT Imaging of Primary Prostate Cancer in Therapy-Naïve Patients. J. Nucl. Med..

[64] Pastorino S., Riondato M., Uccelli L., Giovacchini G., Giovannini E., Duce V., Ciarmiello A. (2020). Toward the Discovery and Development of PSMA Targeted Inhibitors for Nuclear Medicine Applications. Curr. Radiopharm..

[65] Ginn C., Khalili H., Lever R., Brocchini S. (2014). PEGylation and its impact on the design of new protein-based medicines. Future Med. Chem..

[66] Bailon P., Won C.-Y. (2009). PEG-modified biopharmaceuticals. Expert Opin. Drug Deliv..

[67] Nguyen A.T., Kim H.-K. (2023). Recent Developments in PET and SPECT Radiotracers as Radiopharmaceuticals for Hypoxia Tumors. Pharmaceutics.

[68] Baccala A., Sercia L., Li J., Heston W., Zhou M. (2007). Expression of prostate-specific membrane antigen in tumor-associated neovasculature of renal neoplasms. Urology.

[69] Morgantetti G., Ng K.L., Samaratunga H., Rhee H., Gobe G.C., Wood S.T. (2019). Prostate specific membrane antigen (PSMA) expression in vena cava tumour thrombi of clear cell renal cell carcinoma suggests a role for PSMA-driven tumour neoangiogenesis. Transl. Androl. Urol..

