# Supplementary material for: Pharmacokinetic-Modifying Linker Design Modulates Performance of Multivalent PSMA/GRPR-Cell-Targeting 177Lu Radiopharmaceuticals
Source: Cancers (Basel). 2026 Oct 9;18(20):3252. doi: 10.3390/cancers18203252 (PMC13702766; doi:10.3390/cancers18203252)
Supplement: Supplementary file 1 [file cancers-18-03252-s001.zip › cancers-4485547-supplementary.pdf]

Supplemental Data

PSMA-6AHX-[^{177}Lu]-DOTA-CP-BBN								
	PC3-PIP xenografted models				PC3 xenografted models			
**Organ**	**1 h**	**4 h**	**4 h (block)**	**24 h**	**1 h**	**4 h**	**4 h (block)**	**24 h**
**Blood**	0.67 ± 0.22	0.02 ± 0.01	0.02 ± 0.01	0.00 ± 0.00	0.37 ± 0.15	0.02 ± 0.02	0.02 ± 0.02	0.00 ± 0.00
**Heart**	0.26 ± 0.09	0.02 ± 0.01	0.06 ± 0.08	0.01 ± 0.01	0.17 ± 0.07	0.03 ± 0.02	0.09 ± 0.13	0.01 ± 0.00
**Lung**	0.63 ± 0.21	0.12 ± 0.14	0.08 ± 0.07	0.03 ± 0.01	0.39 ± 0.17	0.11 ± 0.09	0.22 ± 0.07	0.06 ± 0.03
**Liver**	0.44 ± 0.20	0.16 ± 0.03	0.31 ± 0.36	0.08 ± 0.01	0.26 ± 0.10	0.15 ± 0.05	0.26 ± 0.10	0.09 ± 0.02
**Spleen**	1.45 ± 1.89	0.13 ± 0.06	0.10 ± 0.07	0.07 ± 0.03	0.34 ± 0.19	0.12 ± 0.03	0.23 ± 0.14	0.09 ± 0.04
**Stomach**	0.80 ± 0.19	0.16 ± 0.16	0.08 ± 0.04	0.02 ± 0.01	0.81 ± 0.30	0.10 ± 0.03	0.10 ± 0.05	0.02 ± 0.01
**L Int**	0.33 ± 0.20	0.39 ± 0.17	0.37 ± 0.27	0.25 ± 0.17	0.21 ± 0.11	0.38 ± 0.14	0.07 ± 0.03	0.13 ± 0.04
**Sm Int**	0.93 ± 0.29	1.32 ± 2.07	0.22 ± 0.15	0.04 ± 0.03	0.68 ± 0.07	0.76 ± 0.87	0.52 ± 0.36	0.02 ± 0.01
**Kidney**	16.30 ± 4.94	4.05 ± 0.69	12.66 ± 1.81	0.96 ± 0.29	8.73 ± 3.44	4.00 ± 1.48	8.33 ± 4.24	0.81 ± 0.12
**Urine***	65.71 ± 20.44	94.12 ± 2.40	89.17 ± 10.78	98.50 ± 0.59	84.42 ± 7.12	86.28 ± 15.19	89.45 ± 9.64	97.36 ± 0.64
**Muscle**	0.10 ± 0.04	0.01 ± 0.00	0.01 ± 0.01	0.00 ± 0.00	0.08 ± 0.06	0.02 ± 0.03	0.03 ± 0.01	0.01 ± 0.01
**Bone**	0.12 ± 0.07	0.01 ± 0.02	0.00 ± 0.02	0.01 ± 0.02	0.13 ± 0.06	0.10 ± 0.17	0.03 ± 0.01	0.03 ± 0.03
**Brain**	0.02 ± 0.01	0.00 ± 0.00	0.00 ±0.00	0.00 ± 0.00	0.02 ± 0.00	0.00 ± 0.00	0.02 ± 0.01	0.00 ± 0.00
**Pancreas**	3.98 ± 1.60	0.40 ± 0.16	0.48 ± 0.27	0.16 ± 0.04	3.25 ± 1.14	0.31 ± 0.11	0.26 ± 0.20	0.15 ± 0.02
**Carcass**	1.68 ± 1.34	0.07 ± 0.02	0.36 ± 0.62	0.03 ± 0.01	0.48 ± 0.36	0.61 ± 1.10	0.35 ± 0.52	0.02 ± 0.00
**Tumor**	5.10 ± 1.57	2.36 ± 0.31	1.75 ± 0.79	0.54 ± 0.21	10.88 ± 2.17	11.14 ± 2.35	5.05 ± 2.35	4.93 ± 0.89
*value represented in Avg %Dose ± SD (n=4). All other values in %ID/g ± SD (n=4)								

Supplemental Table A. *Biodistribution results of PSMA-6AHX-[^{177}Lu]-DOTA-CP-BBN in PC3 and PC3-PIP xenografted mice at 1h, 4 h, 4 h block, and 24 h p.i.*

2PSMA-6AHX-[^{177}Lu]-DOTA-8AOC-BBN								
	PC3-PIP xenografted models				PC3 xenografted models			
**Organ**	**1 h**	**4 h**	**4 h (block)**	**24 h**	**1 h**	**4 h**	**4 h (block)**	**24 h**
**Blood**	1.28 ± 0.19	0.06 ± 0.02	0.04 ± 0.01	0.00 ± 0.00	0.98 ± 0.10	0.03 ± 0.00	0.12 ± 0.07	0.00 ± 0.00
**Heart**	0.47 ± 0.03	0.04 ± 0.01	0.04 ± 0.01	0.01 ± 0.01	0.37 ± 0.08	0.03 ± 0.00	0.22 ± 0.06	0.02 ± 0.00
**Lung**	2.90 ± 2.54	0.19 ± 0.06	0.16 ± 0.03	0.17 ± 0.17	0.90 ± 0.12	0.15 ± 0.05	4.87 ± 2.02	0.07 ± 0.02
**Liver**	0.91 ± 0.11	0.16 ± 0.02	0.17 ± 0.02	0.11 ± 0.01	0.73 ± 0.05	0.19 ± 0.02	0.93 ± 0.38	0.12 ± 0.00
**Spleen**	3.69 ± 1.50	0.41 ± 0.06	0.13 ± 0.05	0.05 ± 0.07	2.66 ± 0.44	0.22 ± 0.04	1.92 ± 0.75	0.09 ± 0.03
**Stomach**	1.86 ± 0.91	0.31 ± 0.08	0.30 ± 0.12	0.04 ± 0.02	1.14 ± 0.23	0.22 ± 0.04	0.86 ± 1.02	0.03 ± 0.00
**L Int**	0.85 ± 0.15	1.36 ± 0.66	1.48 ± 0.89	0.43 ± 0.25	0.65 ± 0.05	1.16 ± 0.36	0.72 ± 0.61	0.51 ± 0.27
**Sm Int**	2.15 ± 0.39	0.60 ± 0.69	0.44 ± 0.23	0.08 ± 0.03	1.74 ± 0.31	0.29 ± 0.04	0.97 ± 0.72	0.08 ± 0.07
**Kidney**	79.97 ± 10.94	13.03 ± 4.43	21.56 ± 2.27	1.84 ± 0.32	75.41 ± 20.35	8.67 ± 0.70	35.00 ± 24.36	1.39 ± 0.20
**Urine***	38.01 ± 9.60	89.33 ± 3.79	89.52 ± 2.65	97.50 ± 0.27	52.69 ± 5.72	92.64 ± 0.25	61.46 ± 19.08	97.58 ± 0.43
**Muscle**	0.16 ± 0.03	0.01 ± 0.00	0.01 ± 0.00	0.00 ± 0.00	0.15 ± 0.02	0.01 ± 0.00	0.06 ± 0.04	0.01 ± 0.00
**Bone**	0.15 ± 0.07	0.04 ± 0.02	0.02 ± 0.01	0.02 ± 0.02	0.13 ± 0.02	0.01 ± 0.01	0.12 ± 0.11	0.00 ± 0.01
**Brain**	0.04 ± 0.01	0.01 ± 0.01	0.01 ± 0.00	0.00 ± 0.00	0.03 ± 0.00	0.01 ± 0.00	0.07 ± 0.02	0.00 ± 0.00
**Pancreas**	13.49 ± 8.37	0.57 ± 0.07	0.72 ± 0.20	0.16 ± 0.07	13.48 ± 4.62	0.44 ± 0.14	3.21 ± 3.09	0.10 ± 0.07
**Carcass**	1.51 ± 0.37	0.23 ± 0.20	0.11 ± 0.10	0.03 ± 0.01	0.84 ± 0.19	0.09 ± 0.04	1.50 ± 1.07	0.02 ± 0.00
**Tumor**	11.15 ± 1.42	6.37 ± 0.64	3.63 ± 0.89	2.02 ± 0.33	12.72 ± 2.96	9.52 ± 2.59	3.12 ± 0.50	4.96 ± 0.32

***value represented in Avg %Dose ± SD (n=4). All other values in %ID/g ± SD (n=4)**

Supplemental Table B. *Biodistribution results of 2PSMA-6AHX-[^{177}Lu]-DOTA-CP-BBN* in PC3 and PC3-PIP xenografted mice *at 1h, 4 h, 4 h block, and 24 h p.i.*

2PSMA-PEG$_4$-[^{177}Lu]-DOTA-8AOC-BBN								
	PC3-PIP xenografted models				PC3 xenografted models			
**Organ**	**1 h**	**4 h**	**4 h (block)**	**24 h**	**1 h**	**4 h**	**4 h (block)**	**24 h**
**Blood**	2.09 ± 1.27	0.10 ± 0.04	0.07 ± 0.01	0.01 ± 0.00	0.53 ± 0.16	0.02 ± 0.00	0.01 ± 0.00	0.00 ± 0.00
**Heart**	0.45 ± 0.13	0.03 ± 0.01	0.03 ± 0.01	0.02 ± 0.01	0.25 ± 0.03	0.03 ± 0.00	0.05 ± 0.00	0.02 ± 0.01
**Lung**	0.89 ± 0.10	0.12 ± 0.01	0.53 ± 0.21	0.38 ± 0.22	0.70 ± 0.17	0.17 ± 0.08	0.48 ± 0.06	0.30 ± 0.06
**Liver**	0.55 ± 0.05	0.16 ± 0.04	0.18 ± 0.02	0.11 ± 0.02	0.46 ± 0.11	0.13 ± 0.01	1.70 ± 2.99	0.12 ± 0.01
**Spleen**	2.76 ± 0.93	0.28 ± 0.11	0.33 ± 0.05	0.08 ± 0.04	2.09 ± 1.63	0.17 ± 0.02	0.26 ± 0.13	0.13 ± 0.02
**Stomach**	0.97 ± 0.23	0.18 ± 0.08	0.26 ± 0.09	0.03 ± 0.01	1.30 ± 0.48	0.27 ± 0.20	0.05 ± 0.11	0.02 ± 0.02
**L Int**	0.57 ± 0.15	1.28 ± 0.61	1.27 ± 0.33	0.14 ± 0.03	0.72 ± 0.43	1.10 ± 0.44	0.42 ± 0.13	0.09 ± 0.02
**Sm Int**	1.72 ± 0.47	0.27 ± 0.06	0.45 ± 0.13	0.03 ± 0.01	1.24 ± 0.20	0.65 ± 0.50	0.19 ± 0.11	0.03 ± 0.01
**Kidney**	83.67 ± 12.02	18.63 ± 6.17	22.27 ± 5.87	1.75 ± 0.33	69.11 ± 47.47	10.22 ± 0.69	12.09 ± 3.44	1.18 ± 0.17
**Urine***	45.43 ± 7.57	89.25 ± 2.37	89.91 ± 1.63	97.64 ± 0.43	61.50 ± 15.38	90.52 ± 1.68	94.03 ± 0.90	97.12 ± 0.55
**Muscle**	0.15 ± 0.01	0.01 ± 0.01	0.01 ± 0.00	0.00 ± 0.00	0.11 ± 0.01	0.01 ± 0.00	0.02 ± 0.02	0.00 ± 0.00
**Bone**	0.15 ± 0.03	0.01 ± 0.01	0.00 ± 0.01	0.01 ± 0.01	0.09 ± 0.02	0.02 ± 0.02	0.02 ± 0.02	0.00 ± 0.01
**Brain**	0.04 ± 0.00	0.01 ± 0.00	0.01 ± 0.00	0.00 ± 0.00	0.02 ± 0.01	0.01 ±0.00	0.01 ± 0.00	0.00 ± 0.00
**Pancreas**	10.87 ± 3.52	0.69 ± 0.41	0.70 ± 0.22	0.13 ± 0.04	7.02 ± 3.86	0.31 ± 0.02	0.13 ± 0.04	0.10 ± 0.06
**Carcass**	1.76 ± 0.35	0.11 ± 0.03	0.09 ± 0.02	0.03 ± 0.01	0.62 ± 0.32	0.08 ± 0.05	0.07 ± 0.04	0.03 ± 0.01
**Tumor**	4.03 ± 0.75	3.30 ± 0.55	2.29 ± 0.67	1.05 ± 0.13	12.54 ± 2.90	9.63 ± 1.03	2.47 ± 0.95	4.86 ± 0.76

***value represented in Avg %Dose ± SD (n=4). All other values in %ID/g ± SD (n=4)**

Supplemental Table C. *Biodistribution results of 2PSMA-PEG$_4$-[^{177}Lu]-DOTA-CP-BBN* in PC3 and PC3-PIP xenografted mice *at 1h, 4 h, 4 h block, and 24 h p.i.*
